# A review and evaluation of doubly robust approaches for estimating average treatment effects

**DOI:** 10.3758/s13428-026-02999-x

**Published:** 2026-04-23

**Authors:** Jingyu Zhang, Oliver Lüdtke, Alexander Robitzsch

**Affiliations:** 1 IPN - Leibniz Institute for Science and Mathematics Education, Olshausenstraße 62, 24118 Kiel, Germany; 2 Centre for International Student Assessment, Kiel, Germany

**Keywords:** Average treatment effect, Causal inference, Outcome regression, Propensity scores, Doubly robust

## Abstract

In nonexperimental studies, obtaining an unbiased estimate of the average treatment effect (ATE) typically requires two key assumptions: that all relevant covariates are measured (i.e., no unmeasured confounding) and that the statistical model used for covariate adjustment is correctly specified. Two common approaches for adjustment are specifying an outcome model and propensity score weighting. To mitigate bias from model misspecification, doubly robust methods combine both approaches, ensuring unbiased ATE estimates if either the outcome model or the propensity score model is correctly specified. In this study, we review four doubly robust methods that have received considerable attention in the methodological literature but remain underutilized in psychological research: augmented inverse probability weighting, regression weighted by the inverse propensity score, regression incorporating the inverse propensity score as a covariate, and calibrated propensity score weighting. Using two simulation studies, we compare these methods with regression estimation and inverse probability weighting estimators. Our results suggest that doubly robust methods—particularly regression weighted by the inverse propensity score—offer greater protection against bias from model misspecification across various data-generating scenarios. We also discuss practical considerations for implementing doubly robust methods, including weight normalization, propensity score truncation, and potential efficiency losses due to overfitting. The different methods for estimating the ATE are illustrated in a data example.

In psychological research, nonexperimental data are frequently used to assess the effect of an exposure or treatment variable on an outcome of interest. For instance, in educational psychology, researchers examine whether private tutoring enhances students’ academic performance. Similarly, in developmental psychology, studies investigate the potential negative effects of substance abuse (e.g., adolescent marijuana use) on adult outcomes (e.g., depression). From a causal inference perspective, researchers often rely on the unconfoundedness assumption, which requires that all relevant confounders—that is, the covariates $\boldsymbol{X}$ affecting both the treatment *T* and the outcome *Y*—are measured (Ding, 2024; Hernán & Robins, 2020; Morgan & Winship, 2015). Crucially, even when unconfoundedness holds because all relevant confounders are measured, valid causal inference still depends on how these covariates are incorporated into the statistical analysis—that is, on correct model specification. For a binary treatment variable, two fundamental strategies are commonly used to adjust for confounding by measured covariates $\boldsymbol{X}$ (Keele et al., 2025; Keller & Branson, 2024). The first uses an outcome model: the outcome is regressed on covariates separately for the treatment and control groups to obtain predicted values, and the average difference in these predictions is taken as the treatment effect. This approach therefore depends on correctly specifying the relationship between the outcome and covariates. In contrast, propensity score methods model the relationship between the treatment and covariates (treatment model) by estimating the propensity score—the conditional probability of receiving treatment given the covariates—typically via logistic regression (Rosenbaum & Rubin, 1983). The propensity score methods aim to achieve covariate balance between treatment and control groups, often through reweighting based on functions of the propensity score, to facilitate unbiased estimation of the treatment effect (Austin, 2011; Hirano & Imbens, 2001).

However, both approaches yield unbiased estimates of the treatment effect only if their respective models are correctly specified, meaning they accurately approximate the true data-generating mechanism. In practice, this is challenging because the true relationships between the outcome and covariates (in the outcome model) and between the treatment and covariates (in the propensity score model) are typically unknown. Doubly robust estimation methods combine both approaches and offer a key advantage: They produce unbiased estimates if either the outcome model or the propensity score model is correctly specified (Bang & Robins, 2005). As a result, they are supposed to be more robust to model misspecification than methods that rely on a single model, effectively giving researchers “two chances to get it right” (Hernán & Robins, 2020, p. 176).

In the present study, we review four doubly robust estimation approaches that received considerable attention in the methodological literature (Ding, 2024; Kang & Schafer, 2007; Robins et al., 2007; Schafer & Kang, 2008) but have been only rarely applied in psychological research: augmented inverse probability weighting (AIPW; Scharfstein et al., 1999), regression weighted by the inverse of the propensity score (Kang & Schafer, 2007), regression with the inverse of the propensity score as a clever covariate (van der Laan & Rose, 2011), and calibrated propensity score weighting (Hainmueller, 2012; Yang, 2018). In our review, we clarify for each method why it provides unbiased estimates of the treatment effect if either the outcome or treatment model is correctly specified. Using two simulation studies, we compare the four doubly robust estimation methods with regression estimation (Schafer & Kang, 2008) and the inverse probability weighting (IPW) estimator under different data-generating scenarios that are typical in psychological research. Our primary focus is to compare the efficiency of the four doubly robust estimators when at least one model is correctly specified. Additionally, we assess whether these estimators provide more accurate treatment effect estimates than the two single-model approaches—regression estimation and IPW—under various scenarios of model misspecification. Furthermore, we examine three key practical considerations. First, we assess whether truncating extreme propensity score weights stabilizes estimator performance. Second, we evaluate refined versions of the AIPW and IPW estimators that use normalized weights (Hájek, 1971). Third, we investigate potential efficiency losses caused by overly complex model specifications, such as including quadratic effects when the true data-generating process only involves linear covariate effects. Before proceeding, we emphasize that our focus is on model misspecification and that we assume throughout that all necessary confounders are measured (i.e., the unconfoundedness assumption).

The article is structured as follows. We begin by introducing the basic setup, including notation and the potential outcomes framework. We define the average treatment effect (ATE), and discuss its identification using observational data, along with the necessary assumptions. Additionally, we describe the parametric specification of nuisance models, such as outcome and propensity score models, which are integral to the ATE estimators. Next, we present ATE estimators, including two single-model estimators and four doubly robust estimators. We explain their mechanics, derive bias formulas under nuisance model misspecification, and highlight the doubly robust properties of the doubly robust estimators. We then report results from two simulation studies. In Study 1, we compare the six estimators in terms of bias and precision across different data-generating mechanisms, particularly examining the robustness of doubly robust estimators under model misspecification. We also explore additional factors, such as propensity score truncation, weight normalization, and model specification choices (linear vs. quadratic nuisance models). In Study 2, we assess whether the key findings from Study 1 generalize to the well-known data-generating mechanism introduced by Kang and Schafer (2007). Following the simulations, we demonstrate the application of these methods in an empirical example. Finally, we summarize our findings, discuss the study’s limitations, and outline directions for future research.

## Average treatment effect

We consider a binary treatment variable *T*, where $T = 1$ indicates treatment and $T = 0$ indicates control. In the potential outcomes framework (Hernán & Robins, 2020; Imbens & Rubin, 2015), each person has two potential outcomes: *Y*(1) represents the potential outcome under the treatment condition, and *Y*(0) represents the potential outcome under the control condition. We further assume that the observed outcome *Y* corresponds to the potential outcome under the observed treatment status

1 $$\begin{aligned} Y = T Y(1) + (1-T) Y(0) . \end{aligned}$$

This assumption is known as the consistency assumption (Hernán & Robins, 2020) and connects the potential outcomes to the observed data (Ding, 2024).

The ATE is defined as

2 $$\begin{aligned} \tau = \textsf{E}\left[ Y(1) - Y(0) \right] , \end{aligned}$$

which represents the expected difference in potential outcomes between the treatment and control conditions. However, since no individual can be observed under both treatment and control conditions simultaneously, the ATE is not identified without additional assumptions (Holland, 1986). In nonexperimental research, this challenge is often addressed by conditioning on a set of observed covariates, assuming that the potential outcomes *Y*(1) and *Y*(0) are independent of the treatment assignment *T* given the covariates $\boldsymbol{X}$, that is

3 $$\begin{aligned} \textsf {P}\left( Y(t), T \mid \boldsymbol{X} \right) = \textsf {P}\left( Y(t) \mid \boldsymbol{X} \right) \textsf {P}\left( T \mid \boldsymbol{X} \right) \text { for } t = 0, 1 . \end{aligned}$$

This assumption, commonly known as ignorability (Rubin, 1978), is also referred to as unconfoundedness, conditional independence, or selection on observables (Hernán & Robins, 2020; Imbens, 2004; Morgan & Winship, 2015). It is important to recognize that ignorability is a strong assumption and should not be taken lightly. In practical applications, it implies that all relevant covariates necessary to satisfy Eq. (3) are observed. The selection of these covariates should be guided by subject-matter expertise rather than relying solely on statistical modeling techniques. However, even experts may disagree on whether all relevant confounders have been adequately measured in a given application. A key strength of the causal inference framework is that it compels researchers to critically evaluate potential confounding variables and acknowledge the strong assumptions required for causal conclusions (Hernán & Robins, 2020).

Moreover, we consider the propensity score (Hahn, 1998; Rosenbaum & Rubin, 1983)

4 $$\begin{aligned} \pi (\boldsymbol{X}) = \textsf {P}(T = 1 \mid \boldsymbol{X}), \end{aligned}$$

which summarizes the covariates as the conditional probability of receiving treatment given $\boldsymbol{X}$. A key requirement for identifying the ATE is that all propensity scores $\pi (\boldsymbol{X})$ lie strictly between 0 and 1. This condition, known as the positivity assumption, ensures that for every possible value $\boldsymbol{x}$ of the covariates, there is some variation in treatment assignment. Under the assumptions of consistency, ignorability, and positivity, we can consider two strategies for identifying the ATE.

### Identification of average treatment effect

First, the ATE can be identified as (Ding, 2024; Rosenbaum & Rubin, 1983)

5 $$\begin{aligned} \tau = \textsf{E}\left[ Y(1) - Y(0) \right] = \textsf{E}\left[ \mu _1(\boldsymbol{X}) - \mu _0(\boldsymbol{X}) \right] , \end{aligned}$$

where the conditional expectations $\mu _t(\boldsymbol{X}) = \textsf{E}(Y \mid T = t, \boldsymbol{X})$ for $t=0,1$ describe the mean of *Y* given *T* and $\boldsymbol{X}$, and the outer expectation in Eq. (5) is taken with respect to the marginal distribution of the covariates $\boldsymbol{X}$. Note that the positivity assumption is implicitly required in Eq. (5) because the conditional expectations $\mu _t(\boldsymbol{X})$ are only well-defined if $0< \pi (\boldsymbol{X}) < 1$ (Ding, 2024). In practice, this identification strategy requires estimating two regression functions: one for the treatment group ($T=1$) and one for the control group ($T=0$).

Another strategy for identifying the ATE relies directly on the propensity score

6 $$\begin{aligned} \tau = \textsf{E}\left[ \frac{TY}{\pi (\boldsymbol{X})} - \frac{(1-T)Y}{1-\pi (\boldsymbol{X})} \right] . \end{aligned}$$

This expression, also referred to as the IPW formula (Ding, 2024), motivates various propensity score weighting approaches. In this strategy, positivity is a crucial technical requirement, as it prevents division by zero when the propensity score appears in the denominator.

In practice, parametric models with linearity assumptions are often used to model the relationship between covariates and the outcome (i.e., outcome model $\mu _t(\boldsymbol{X})$) or between covariates and treatment (i.e., propensity score model $\pi (\boldsymbol{X})$). These are called “nuisance” models because their primary purpose is to adjust for confounding and improve estimation efficiency, rather than to assess the effects of individual covariates.

### Estimation of nuisance models

For the first identification strategy (Eq. (5)) with a continuous outcome variable, two linear regression models are assumed to describe the relationship between the covariates $\boldsymbol{X}$ and the outcome *Y* in the treatment and control groups

7 $$\begin{aligned} \mu _1( \boldsymbol{X}, \boldsymbol{\beta }_1) = \boldsymbol{\beta }_1^\top \boldsymbol{X} \text { and } \mu _0( \boldsymbol{X}, \boldsymbol{\beta }_0) = \boldsymbol{\beta }_0^\top \boldsymbol{X}. \end{aligned}$$

Based on empirical data $(y_i, t_i, \boldsymbol{x}_i)$ for a sample of individuals $i = 1,\dots , N$, the estimated regression coefficients $\hat{\boldsymbol{\beta }}_1$ and $\hat{\boldsymbol{\beta }}_0$ satisfy the following moment conditions in the treatment and control group

8 $$\begin{aligned} \sum _{i=1}^N t_i \boldsymbol{x}_i (y_i - \hat{\boldsymbol{\beta }}_1^\top \boldsymbol{x}_i) = 0 \text { and } \sum _{i=1}^N (1-t_i) \boldsymbol{x}_i (y_i - \hat{\boldsymbol{\beta }}_0^\top \boldsymbol{x}_i) = 0. \end{aligned}$$

Note that fitting separate regression models in the group of treated $(t_i=1)$ and the group of untreated $(t_i=0)$ persons automatically accounts for all treatment-by-covariate interactions (Schafer & Kang, 2008). Furthermore, the covariate vector $\boldsymbol{x}_i$ can also include transformed covariates, such as multiplicative interactions $(x_{1i}x_{2i})$ or squared terms $(x_{1i}^2)$.

Based on the second identification strategy (Eq. (6)), propensity score methods often rely on logistic regression to model the relationship between the covariates and the treatment

9 $$\begin{aligned} \pi ( \boldsymbol{X}, \boldsymbol{\alpha } ) = {\text {expit}}(\boldsymbol{\alpha }^\top \boldsymbol{X}), \end{aligned}$$

where ${\text {expit}}(y) = \exp (y) / \{ 1+\exp (y) \}$ is the logistic function. With empirical data, the logistic regression coefficients $\hat{\boldsymbol{\alpha }}$ are obtained with maximum likelihood estimation and fulfill the first-order condition

10 $$\begin{aligned} \sum _{i=1}^N \boldsymbol{x}_i \{t_i - {\text {expit}}( \hat{\boldsymbol{\alpha }}^\top \boldsymbol{x}_i )\} = 0. \end{aligned}$$

Even if all relevant covariates $\boldsymbol{X}$ required to satisfy the ignorability assumption are measured, it is essential that either the outcome-covariate relationship (see Eq. (8)) or the treatment-covariate relationship (see Eq. (10)) is correctly specified (Keele et al., 2025). This includes ensuring that important nonlinearities, such as quadratic effects, are accounted for (e.g., by including squared terms of covariates when necessary, see Dehejia & Wahba, 2002; Millimet & Tchernis, 2009; Shah et al., 2005; Smith & Todd, 2005). In the next section, we review four doubly robust estimators of the ATE that remain unbiased if either of the two nuisance models—the outcome model or the treatment model—is correctly specified, though not necessarily both. Before doing so, we briefly discuss why single-modeling approaches yield biased estimates if their corresponding nuisance models are misspecified.

## Estimation of average treatment effects

We begin with two single-modeling approaches: regression estimation and the IPW estimator. Next, we explore four dual-modeling approaches, where only one of the two models needs to be correctly specified. It is important to emphasize that, in the following discussion, we assume the ignorability condition holds (i.e., there is no bias from unmeasured confounding) and focus on the correct specification of the nuisance models. Accordingly, we are concerned only with bias arising from model misspecification and assume that all necessary covariates are measured and included in the analysis.

### Regression estimation

Regression estimation is a method used to estimate the ATE by modeling the relationship between the outcome *Y* and the covariates $\boldsymbol{X}$. It is motivated by the identification result in Eq. (5) and involves two separate regression models that are estimated for the groups of treated (i.e., $T = 1$) and untreated (i.e., $T = 0$) persons (see Eq. (8)). An estimate of the ATE is then obtained by averaging the predictions from the two regression models across persons in the sample

11 $$\begin{aligned} \hat{\tau }_{\text {Reg}} = N^{-1} \sum _{i=1}^N \left\{ \mu _1(\boldsymbol{X}_i, \hat{\boldsymbol{\beta }}_1) - \mu _0(\boldsymbol{X}_i, \hat{\boldsymbol{\beta }}_0) \right\} , \end{aligned}$$

where $\mu _1(\boldsymbol{X}_i, \hat{\boldsymbol{\beta }}_1)$ and $\mu _0(\boldsymbol{X}_i, \hat{\boldsymbol{\beta }}_0)$ are the predicted outcomes for person *i* from the estimated regression in the treatment and control groups (Rubin, 1977; Schafer & Kang, 2008). In the causal inference literature, the regression estimation approach is also labeled as parametric g-formula (Hernán & Robins, 2020; Robins et al., 1992), standardization with a parametric outcome model (Brumback, 2022) or generalized ANCOVA (Mayer et al., 2016). However, if the regression models are misspecified, the estimated ATE ($\hat{\tau }_{\text {Reg}}$) from regression estimation will, in general, be biased. The bias of $\hat{\tau }_{\text {Reg}}$ can be derived as

12 $$\begin{aligned} \textsf {Bias}( \hat{\tau }_{\text {Reg}})= &  -\textsf{E}\left[ \left\{ 1-\pi (\boldsymbol{X}) \right\} \left\{ \mu _1(\boldsymbol{X}) - \mu _1( \boldsymbol{X}, \hat{\boldsymbol{\beta }}_1) \right\} \right. \nonumber \\ &  \left. + \pi (\boldsymbol{X}) \left\{ \mu _0(\boldsymbol{X}) - \mu _0( \boldsymbol{X}, \hat{\boldsymbol{\beta }}_0) \right\} \right] . \end{aligned}$$

As can be seen, the bias of the estimator obtained from regression estimation is a function of the misspecification of the regression models multiplied by functions of the propensity scores. The bias formulas are further exemplified in the Section “Illustrating the effects of misspecification with a single covariate”^1^.

### Inverse probability weighting (IPW)

The identification strategy in Eq. (6) motivates the following Horvitz–Thompson (HT; Horvitz & Thompson, 1952) estimator of the ATE

13 $$\begin{aligned} \hat{\tau }_\text {IPW}^\text {HT} = N^{-1} \sum _{i=1}^N \frac{T_i Y_i}{\pi (\boldsymbol{X}_i, \hat{\boldsymbol{\alpha }})} - N^{-1} \sum _{i=1}^N \frac{ (1-T_i) Y_i}{1-\pi (\boldsymbol{X}_i, \hat{\boldsymbol{\alpha }})} . \end{aligned}$$

The HT estimator is an IPW estimator, meaning that it reweights observations based on their propensity scores. The weights $W_i = W_i ( T_i, \boldsymbol{X} _i )$ for each unit are the inverse of the probability of receiving its observed treatment status, that is $W_i = 1 / \pi ( \boldsymbol{X}_i, \hat{\boldsymbol{\alpha }})$ for persons in the treatment ($T_i = 1$), and $W_i = 1 / \left( 1 - \pi ( \boldsymbol{X}_i, \hat{\boldsymbol{\alpha }}) \right)$ for persons in the control group ($T_i = 0$). As a result, individuals who are very unlikely to be assigned to treatment are upweighted in the treatment condition and downweighted in the control condition, and vice versa. Formally, the weights can be compactly written as

14 $$\begin{aligned} W_i = \frac{T_i}{ \pi ( \boldsymbol{X}_i, \hat{\boldsymbol{\alpha }}) } + \frac{1-T_i}{ 1-\pi ( \boldsymbol{X}_i, \hat{\boldsymbol{\alpha }}) }. \end{aligned}$$

The IPW estimator in Eq. (13) can be rephrased as

15 $$\begin{aligned} \hat{\tau }_{\text {IPW}}^{\text {HT}} = N^{-1} \sum _{i=1}^N W_i T_i Y_i - N^{-1} \sum _{i=1}^N W_i (1-T_i) Y_i. \end{aligned}$$

The IPW estimator removes confounding by creating a pseudopopulation where the treatment assignment is independent of the measured covariates. However, if the propensity score model is misspecified, the estimator in Eq. (13) may yield biased estimates of the ATE

16 $$\begin{aligned} \textsf {Bias}(\hat{\tau }_\text {IPW}^\text {HT}) = \textsf{E}\left[ \left\{ \pi (\boldsymbol{X}) - \pi (\boldsymbol{X}, \hat{\boldsymbol{\alpha }}) \right\} \left\{ \frac{\mu _1(\boldsymbol{X})}{\pi (\boldsymbol{X}, \hat{\boldsymbol{\alpha }})} {+} \frac{\mu _0(\boldsymbol{X})}{1-\pi (\boldsymbol{X}, \hat{\boldsymbol{\alpha }})} \right\} \right] . \end{aligned}$$

As can be seen, the bias of $\hat{\tau }_\text {IPW}^\text {HT}$ is a function of the misspecification of the propensity score model and the sum of the conditional expectations in the treatment and the control group, weighted by the inverse of the estimated propensity scores.

The propensity score estimator in Eq. (13) weights the observations by the inverse of the estimated propensity score. If the true propensity scores were used, the sum of the weights should equal the total sample size in the treatment and control groups, that is, $\sum _{i=1}^N W_i T_i = N$, and $\sum _{i=1}^N W_i (1-T_i) = N$. However, in the case of a misspecified propensity score model and of small to moderate samples, the sum of the weights can substantially differ between the two groups (Hirano & Imbens, 2001; Imbens, 2004). To address this issue, it has been proposed to normalize the weights when estimating the ATE

17 $$\begin{aligned} \hat{\tau }_\text {IPW}^\text {Hajek} = \frac{ \displaystyle \sum _{i=1}^N W_i T_i Y_i}{ \displaystyle \sum _{i=1}^N W_i T_i} - \frac{ \displaystyle \sum _{i=1}^N W_i (1-T_i) Y_i}{ \displaystyle \sum _{i=1}^N W_i (1-T_i)}, \end{aligned}$$

which is commonly known as the Hajek estimator (Hájek, 1971). This estimator is widely recommended in the literature because it generally exhibits lower variance than the IPW estimator with HT weights ($\hat{\tau }_\text {IPW}^\text {HT}$) (Imai & Ratkovic, 2014; Khan & Ugander, 2023; Liu et al., 2016; Luo et al., 2025; Millimet & Tchernis, 2009; Wang et al., 2021). Although its random denominator may introduce a small bias, the Hajek estimator is typically more stable and efficient in most practical scenarios (Gao & Ding, 2023; Särndal et al., 2003). Additionally, unlike the IPW estimator with HT weights, the Hajek estimator remains invariant under a location shift of the outcome (Ding, 2024; Gao & Ding, 2023).

Another challenge for the application of IPW estimators is their tendency to exhibit high variability, particularly in small to moderate samples and when covariate distributions differ substantially between treatment and control groups (Cole & Hernán, 2008). Although treated units with low propensity scores and control units with high propensity scores provide ideal counterfactual comparisons, their estimated probability values can be too extreme (i.e., close to 0 or 1). This leads to inflated inverse probability weights, potentially causing the IPW estimator to become unstable (Ding, 2024). To address this issue, weight truncation has been proposed as a stabilization technique for the IPW estimator. Truncation modifies the estimated propensity score $\pi ( \boldsymbol{x}_i, \hat{\boldsymbol{\alpha }})$ as follows

18 $$\begin{aligned} \max \left[ \epsilon , \min \left\{ \pi (\boldsymbol{x}_i, \hat{\boldsymbol{\alpha }}), 1-\epsilon \right\} \right] , \end{aligned}$$

where any propensity score below $\epsilon$ is set to $\epsilon$, and any score above $1-\epsilon$ is set to $1-\epsilon$. For example, with $\epsilon =0.05$, all estimated propensity scores lie in the interval [0.05, 0.95]. On the one hand, truncation introduces asymptotic bias by artificially distorting the propensity score distribution. On the other hand, it reduces variance by limiting the influence of extreme weights and minimizing the impact of a few non-representative individuals on the effect estimate. This is usually described as a trade-off between bias and variance (Cole & Hernán, 2008; Petersen et al., 2012). In finite samples, accepting a small bias in exchange for a significant reduction in variance often improves estimator performance. However, as the sample size approaches infinity, variance converges to a limit, while truncation-induced bias remains and swamps the benefits of variance reduction (Gruber et al., 2022; Petersen et al., 2012). Therefore, the goal is to select an appropriate truncation level that optimally balances between bias and variance. For example, truncation levels of [0.01, 0.99] and [0.05, 0.95] are usually chosen (Leite et al., 2019; Thoemmes & Ong, 2016). Recent studies suggest that truncation levels can also be determined by data-driven approaches (Gruber et al., 2022; Ju et al., 2019).

### Augmented inverse probability weighting (AIPW)

The augmented inverse probability weighting (Bang & Robins, 2005; Robins et al., 1994; Scharfstein et al., 1999) estimator is a doubly robust approach that combines IPW with an outcome model to estimate the ATE. The basic idea is to improve the efficiency of the traditional IPW estimator, which only incorporates information from the treatment model, by augmenting it with an outcome model. More specifically, the AIPW estimator combines the fitted values of the propensity scores, that is ${\pi (\boldsymbol{X}_i, \hat{\boldsymbol{\alpha }})}$, with the predicted outcomes of the two regression models, that is ${\mu _1(\boldsymbol{X}_i, \hat{\boldsymbol{\beta }}_1) }$ and ${\mu _0(\boldsymbol{X}_i, \hat{\boldsymbol{\beta }}_0) }$, and is written as follows

19 $$\begin{aligned} \hat{\tau }_\text {AIPW}^{\text {HT}}= &  N^{-1} \sum _{i=1}^N \left[ \frac{T_i Y_i }{\pi (\boldsymbol{X}_i, \hat{\boldsymbol{\alpha }})} - \frac{(1-T_i)Y_i }{1-\pi (\boldsymbol{X}_i, \hat{\boldsymbol{\alpha }})} \right] \nonumber \\ &  + N^{-1} \sum _{i=1}^N \left\{ \pi (\boldsymbol{X}_i, \hat{\boldsymbol{\alpha }}) - T_i \right\} \left\{ \frac{ \mu _1(\boldsymbol{X}_i, \hat{\boldsymbol{\beta }}_1) }{\pi (\boldsymbol{X}_i, \hat{\boldsymbol{\alpha }})} + \frac{ \mu _0(\boldsymbol{X}_i, \hat{\boldsymbol{\beta }}_0) }{1-\pi (\boldsymbol{X}_i, \hat{\boldsymbol{\alpha }}) } \right\} . \end{aligned}$$

Note that the first term represents the IPW estimator (with the HT weights; see Eq. (13)), while the second term incorporates predicted outcomes from the two regression models fitted separately in the treatment and control groups. Alternatively, the AIPW estimator can also be written as:

20 $$\begin{aligned} \hat{\tau }_\text {AIPW}^{\text {HT}}= &  N^{-1} \sum _{i=1}^N \left[ \mu _1(\boldsymbol{X}_i, \hat{\boldsymbol{\beta }}_1) - \mu _0(\boldsymbol{X}_i, \hat{\boldsymbol{\beta }}_0) \right] \nonumber \\ &  + N^{-1} \sum _{i=1}^N \left[ \frac{T_i\{Y_i - \mu _1(\boldsymbol{X}_i, \hat{\boldsymbol{\beta }}_1 )\}}{\pi (\boldsymbol{X}_i, \hat{\boldsymbol{\alpha }})} - \frac{(1-T_i)\{Y_i - \mu _0(\boldsymbol{X}_i , \hat{\boldsymbol{\beta }}_0)\}}{1-\pi (\boldsymbol{X}_i, \hat{\boldsymbol{\alpha }})} \right] . \end{aligned}$$

Thus, the AIPW estimator can also be viewed as an extension of regression estimation (see Eq. (11)), incorporating additional terms. These terms are the residuals of the outcomes models, $Y_i - \mu _1(\boldsymbol{X}_i, \hat{\boldsymbol{\beta }}_1)$ and $Y_i - \mu _0(\boldsymbol{X}_i, \hat{\boldsymbol{\beta }}_0)$, which are weighted by the inverse propensity scores, $T_i / \pi (\boldsymbol{X}_i, \hat{\boldsymbol{\alpha }})$ and $(1-T_i) / (1-\pi (\boldsymbol{X}_i, \hat{\boldsymbol{\alpha }}))$, respectively. If the predicted outcomes coincide with the observed outcomes $Y_i$, the residuals become zero, and the additional terms vanish, reducing the AIPW estimator to regression estimation.

In the case that both the outcome and the propensity score model are misspecified, the bias of the AIPW estimator for the ATE is given as (Ding, 2024; Hernán & Robins, 2020)

21 $$\begin{aligned} \textsf {Bias}( \hat{\tau }_\text {AIPW}^{\text {HT}} )= &  \textsf{E}\Bigg [ \left\{ \pi ( \boldsymbol{X} ) - \pi ( \boldsymbol{X} , \hat{ \boldsymbol{\alpha }} ) \right\} \Bigg \{ \frac{ \mu _1 ( \boldsymbol{X} ) - \mu _1 ( \boldsymbol{X} , \hat{\boldsymbol{\beta }}_1) }{ \pi ( \boldsymbol{X} , \hat{ \boldsymbol{\alpha }} ) } \nonumber \\ &  + \frac{ \mu _0 ( \boldsymbol{X} ) - \mu _0 ( \boldsymbol{X} , \hat{\boldsymbol{\beta }}_0) }{ 1 - \pi ( \boldsymbol{X} , \hat{ \boldsymbol{\alpha }} ) } \Bigg \} \Bigg ] . \end{aligned}$$

As can be seen, the bias of the AIPW estimator depends on the misspecification of the propensity score model and of the two regression models. Interestingly, the two errors multiply, potentially resulting in much larger errors (Kang & Schafer, 2007). In his discussion of the bias of the AIPW estimator, Ding (2024, p. 158) writes: “This delicate structure renders the doubly robust estimator possibly doubly fragile when both the propensity score and the outcome models are misspecified.” We return to this issue in the Section “Illustrating the effects of misspecification with a single covariate” and in the two simulation studies, where we assess whether misspecifying both nuisance models can lead doubly robust estimators (e.g., AIPW) to perform poorly.

In order to make the AIPW estimator less sensitive to the influence of extreme weights, the weights in Eq. (20) can also be normalized. This results in the following alternative Hajek form of the AIPW estimator (Kang & Schafer, 2007; Robins et al., 2007)

22 $$\begin{aligned} \hat{\tau }_\text {AIPW}^\text {Hajek}= &  N^{-1} \sum _{i=1}^N \left[ \mu _1(\boldsymbol{X}_i, \hat{\boldsymbol{\beta }}_1) - \mu _0(\boldsymbol{X}_i, \hat{\boldsymbol{\beta }}_0) \right] \nonumber \\ &  + \frac{ \displaystyle \sum _{i=1}^N W_i T_i \{Y_i - \mu _1(\boldsymbol{X}_i, \hat{\boldsymbol{\beta }}_1)\}}{ \displaystyle \sum _{i=1}^N W_i T_i} -\frac{ \displaystyle \sum _{i=1}^N W_i (1-T_i ) \{Y_i - \mu _0(\boldsymbol{X}_i, \hat{\boldsymbol{\beta }}_0)\}}{ \displaystyle \sum _{i=1}^N W_i (1-T_i) } , \end{aligned}$$

where the weights $W_i$ are defined as in Eq. (14). Most introductions to the AIPW estimator focus on the unnormalized (HT) form without mentioning this alternative Hajek form (Funk et al., 2011; Glynn & Quinn, 2010; Kurz, 2022). Only a few simulations included AIPW Hajek, showing that it produces ATE estimates nearly identical to AIPW HT (Chattopadhyay et al., 2020; Khan & Ugander, 2023).

### Regression weighted by the inverse of the propensity score

Regression weighted by the inverse of the propensity scores is another approach to estimate the ATE that combines propensity score weighting with an outcome model (Hirano & Imbens, 2001; Imbens, 2004; Kang & Schafer, 2007; Robins et al., 2007; Schafer & Kang, 2008). This approach involves fitting two regression models (in the treatment and control groups) where each observation is weighted by its inverse propensity score. The structure of this weighted regression estimator of the ATE is similar to regression estimation (see Eq. (11))

23 $$\begin{aligned} \hat{\tau }_{\text {WReg}} = N^{-1} \sum _{i=1}^N \left\{ \mu _1(\boldsymbol{X}_i, \hat{\boldsymbol{\beta }}_1^W) - \mu _0(\boldsymbol{X}_i, \hat{\boldsymbol{\beta }}_0^W) \right\} , \end{aligned}$$

where the coefficients $\hat{\boldsymbol{\beta }}_1^W$ and $\hat{\boldsymbol{\beta }}_0^W$ were estimated using weighted least squares (WLS) with inverse propensity score weights, that is $T_i/\pi (\boldsymbol{X}_i, \hat{\boldsymbol{\alpha }})$ for estimating $\hat{\boldsymbol{\beta }}_1^W$ and $(1-T_i)/(1-\pi (\boldsymbol{X}_i, \hat{\boldsymbol{\alpha }}))$ for estimating $\hat{\boldsymbol{\beta }}_0^W$. More specifically, the regression coefficients fulfill the following moment conditions in the treatment ($t_i=1$) and control groups ($t_i = 0$)

24 $$\begin{aligned} &  \sum _{i=1}^N \frac{t_i}{\pi (\boldsymbol{x}_i, \hat{\boldsymbol{\alpha }}) } \boldsymbol{x}_i \{ y_i - ( \hat{\boldsymbol{\beta }}^W_1 )^\top \boldsymbol{x}_i \} = 0 \text { and }\nonumber \\ &  \sum _{i=1}^N \frac{1-t_i}{1-\pi (\boldsymbol{x}_i, \hat{\boldsymbol{\alpha }}) } \boldsymbol{x}_i \{ y_i - ( \hat{\boldsymbol{\beta }}^W_0 )^\top \boldsymbol{x}_i \} = 0. \end{aligned}$$

By incorporating the inverse of the propensity scores as weights in WLS, individuals contribute differently to the estimation of the regression coefficients. However, it is crucial to note that these weights are used solely to estimate the regression coefficients and are not applied when calculating the predicted outcomes in Eq. (23). Thus, the inverse propensity score weights do not directly determine the ATE, but rather influence its estimation indirectly by affecting the coefficients $\hat{\boldsymbol{\beta }}_1^W$ and $\hat{\boldsymbol{\beta }}_0^W$ in the regression models.

Several scholars have highlighted that regression weighted by the inverse of the propensity score is doubly robust (Ding, 2024; Gabriel et al., 2024; Kang & Schafer, 2007). However, if both the propensity score model and the outcome model are misspecified, $\hat{\tau }_{\text {WReg}}$ produces a biased estimate of the ATE

25 $$\begin{aligned} \textsf {Bias}( \hat{\tau }_{\text {WReg}} )= &  \textsf{E}\Bigg [ \left\{ \pi ( \boldsymbol{X}) - \pi ( \boldsymbol{X}, \hat{\boldsymbol{\alpha }}) \right\} \Bigg \{ \frac{ \mu _1 ( \boldsymbol{X}) - \mu _1 ( \boldsymbol{X}, \hat{\boldsymbol{\beta }}^W_1 ) }{ \pi ( \boldsymbol{X}, \hat{\boldsymbol{\alpha }}) } \nonumber \\ &  +\frac{ \mu _0 ( \boldsymbol{X}) - \mu _0 ( \boldsymbol{X}, \hat{\boldsymbol{\beta }}^W_0 ) }{ 1 - \pi ( \boldsymbol{X}, \hat{\boldsymbol{\alpha }}) } \Bigg \} \Bigg ] . \end{aligned}$$

Interestingly, the bias expression follows the same structure as that of the AIPW estimator (see Eq. (21)): The estimation errors from the propensity score model and the outcome model interact multiplicatively, potentially leading to significantly larger errors when both models are misspecified. Simulation studies suggest that this estimator ($\hat{\tau }_{\text {WReg}}$) slightly outperforms the AIPW estimator (Imai & Ratkovic, 2014; Robins et al., 2007; Schafer & Kang, 2008). However, it was argued that $\hat{\tau }_{\text {WReg}}$ may introduce additional random error and bias, making single-modeling approaches (i.e., regression estimation) preferable when the outcome model is correctly specified (Freedman & Berk, 2008).

There are two additional considerations when applying regression weighted by the inverse of the propensity score. First, weight normalization is not necessary for $\hat{\tau }_{\text {WReg}}$ because regression weighted by the inverse propensity score naturally produces estimators of the Hajek form, ensuring more stable estimation of the ATE (Ding, 2024; Imbens, 2004). Second, as with all propensity score weighting estimators, truncating extreme weights can improve the stability of $\hat{\tau }_{\text {WReg}}$ and mitigate the effects of model misspecification. However, while truncation reduces variance, it may introduce additional bias, potentially undermining the double robustness property.

### Regression with the inverse of the propensity score as a clever covariate

Propensity scores can also serve as additional covariates that are included in the outcome model (Kang & Schafer, 2007; Robins et al., 2007). One specific transformation of the propensity scores is known as the “clever covariate” (Moore & van der Laan, 2009; van der Laan & Rose, 2011) and is given as

26 $$\begin{aligned} H_i = H_i (T_i , \boldsymbol{X} _i ) = \frac{T_i}{\pi (\boldsymbol{X}_i, \hat{\boldsymbol{\alpha }})} - \frac{1-T_i}{1-\pi (\boldsymbol{X}_i, \hat{\boldsymbol{\alpha }})} , \end{aligned}$$

where for an individual *i*, $H_i$ simplifies to $1/\pi (\boldsymbol{X}_i, \hat{\boldsymbol{\alpha }})$ if *i* belongs to the treatment group and $-1/(1-\pi (\boldsymbol{X}_i, \hat{\boldsymbol{\alpha }}))$ if *i* belongs to the control group. In the next step, the residuals from the two outcome models are regressed on $H_i$ in a no-intercept ordinary least-squares (OLS) regression

27 $$\begin{aligned} \widetilde{Y}_i = \phi H_i + \varepsilon _i, \end{aligned}$$

where $\widetilde{Y}_i = T_i \{ Y_i - \mu _1(\boldsymbol{X}_i, \hat{\boldsymbol{\beta }}_1) \} + (1-T_i) \{ Y_i - \mu _0(\boldsymbol{X}_i, \hat{\boldsymbol{\beta }}_0) \}$ represents the residual of the outcome variable, $\phi$ is the coefficient of the clever covariate, and $\varepsilon _i$ is the regression residual. The estimated coefficient of $H_i$ is also referred to as the fluctuation parameter because it provides information about how much to change or fluctuate the initial estimates of the outcome model (Gruber & van der Laan, 2012). The estimated fluctuation parameter $\hat{\phi }$ can be obtained as

28 $$\begin{aligned} \hat{\phi } = \frac{ \displaystyle \sum _{i=1}^N\frac{T_i ( Y _i -\mu _1 ( \boldsymbol{X}_i, \hat{\boldsymbol{\beta }}_1 ) ) }{\pi ( \boldsymbol{X}_i , \hat{\boldsymbol{\alpha }})} - \sum _{i=1}^N\frac{(1-T_i) ( Y_i-\mu _0 ( \boldsymbol{X}_i, \hat{\boldsymbol{\beta }}_0 ) ) }{1 - \pi ( \boldsymbol{X}_i , \hat{\boldsymbol{\alpha }})} }{ \displaystyle \sum _{i=1}^N\frac{T _i}{\pi ( \boldsymbol{X}_i , \hat{\boldsymbol{\alpha }})^2} + \sum _{i=1}^N\frac{1-T _i }{(1-\pi ( \boldsymbol{X}_i , \hat{\boldsymbol{\alpha }}))^2} } . \end{aligned}$$

In the final step, the clever covariate $H_i$ and its estimated fluctuation parameter $\hat{\phi }$ are incorporated into a regression estimator to obtain an estimate of the ATE

29 $$\begin{aligned} \hat{\tau }_\text {Clev} = N^{-1} \sum _{i = 1}^N &  \left[ \mu _1(\boldsymbol{X}_i, \hat{\boldsymbol{\beta }}_1) - \mu _0(\boldsymbol{X}_i, \hat{\boldsymbol{\beta }}_0)\right. \nonumber \\ &  \left. + \frac{\hat{\phi }}{\pi (\boldsymbol{X}_i, \hat{\boldsymbol{\alpha }})} + \frac{\hat{\phi }}{1-\pi (\boldsymbol{X}_i, \hat{\boldsymbol{\alpha }})} \right] . \end{aligned}$$

The resulting estimator can be seen as an augmented regression estimator, where the augmentation term represents the outcome residuals predicted by the clever covariate. By incorporating this adjustment, the clever covariate corrects for residual confounding arising from a misspecified outcome model (Hernán & Robins, 2020).

To derive the bias of $\hat{\tau }_\text {Clev}$, we introduce the quantity $\Delta$ defined as

30 $$\begin{aligned} \Delta = \frac{ \displaystyle \textsf{E}\left[ \frac{1}{\pi ( \boldsymbol{X}, \hat{\boldsymbol{\alpha }})} + \frac{1}{1 - \pi ( \boldsymbol{X}, \hat{\boldsymbol{\alpha }})} \right] }{ \textsf{E}\left[ \displaystyle \frac{\pi ( \boldsymbol{X}) }{\pi ( \boldsymbol{X}, \hat{\boldsymbol{\alpha }})^2} + \frac{1-\pi ( \boldsymbol{X}) }{(1-\pi ( \boldsymbol{X}, \hat{\boldsymbol{\alpha }}))^2} \right] } . \end{aligned}$$

Note that $\Delta =1$ if $\pi (\boldsymbol{X}) = \pi ( \boldsymbol{X}, \hat{\boldsymbol{\alpha }})$. The bias of $\hat{\tau }_\text {Clev}$ for estimating the ATE can then be determined as

31 $$\begin{aligned} \begin{array}{rcl} \textsf {Bias}( \hat{\tau }_\text {Clev} ) & = & \displaystyle \textsf{E}\left[ \left\{ 1 - \frac{ \pi ( \boldsymbol{X}) }{\pi ( \boldsymbol{X}, \hat{\boldsymbol{\alpha }})} \Delta \right\} \{ \mu _1 ( \boldsymbol{X}) -\mu _1 ( \boldsymbol{X}, \hat{\boldsymbol{\beta }}_1 ) \} \right] \\ & & \displaystyle \quad + \textsf{E}\left[ \left\{ 1 - \frac{ 1- \pi ( \boldsymbol{X}) }{1-\pi ( \boldsymbol{X}, \hat{\boldsymbol{\alpha }})} \Delta \right\} \{ \mu _0 ( \boldsymbol{X}) -\mu _0 ( \boldsymbol{X}, \hat{\boldsymbol{\beta }}_0 ) \} \right] . \end{array} \end{aligned}$$

As can be seen, the estimator is doubly robust because the bias vanishes if either the outcome model or the propensity score model is correctly specified (Bang & Robins, 2005; Schuler & Rose, 2017). Furthermore, if both are misspecified, the bias for $\hat{\tau }_\text {Clev}$ follows a similar multiplicative structure as that of the AIPW estimator ($\hat{\tau }_\text {AIPW}^{\text {HT}}$) and regression weighted by the inverse of the propensity score ($\hat{\tau }_{\text {WReg}}$). Simulations confirm the doubly robustness of the estimator (Bang & Robins, 2005; Muñoz & van der Laan, 2012; Pang et al., 2016; Schuler & Rose, 2017). Overall, it performs similarly to the AIPW estimator, but it can outperform AIPW in sparse data settings and in high-dimensional confounding scenarios (Ellul et al., 2024; Gruber & van der Laan, 2010a, b; Luque-Fernandez et al., 2018). A further advantage of $\hat{\tau }_\text {Clev}$ is that it can be combined with non-parametric methods, which may improve performance under model misspecification (Hoffmann, 2023; Pirracchio et al., 2015; Rudolph et al., 2023; Schuler & Rose, 2017). However, performance can deteriorate when propensity scores are highly variable, and the outcome model is affected by extrapolation (Kang & Schafer, 2007; Schafer & Kang, 2008)—settings in which weighted regression may perform more favorably (Robins et al., 2007).

In practical applications, the outcome *Y* is sometimes transformed using the logit function when calculating $\hat{\tau }_\text {Clev}$ (Hoffmann, 2023; Luque-Fernandez et al., 2018; Schuler & Rose, 2017). For bounded outcomes, this transformation helps ensure that predicted values remain within the defined range (Gruber & van der Laan, 2010b). Additionally, it can enhance the performance of $\hat{\tau }_\text {Clev}$ in sparse data settings, particularly when the positivity assumption is nearly violated, leading to extreme propensity score weights. However, this transformation alters the outcome scale and is not strictly necessary (e.g., van der Laan & Rubin, 2006). In this study, we do not apply such a transformation and instead estimate the ATE on the original scale of *Y* (Gruber & van der Laan, 2012). Moreover, $\hat{\tau }_\text {Clev}$ can also be stabilized by truncating extreme weights in the calculation of the clever covariate (see Eq. (26)).

### Calibrated propensity score weighting

The basic idea of propensity score weighting approaches is to create a pseudopopulation in which the treatment indicator *T* is independent of the covariates $\boldsymbol{X}$. In this weighted pseudopopulation, the means of the observed covariates are balanced across the treatment and control conditions. However, if the propensity score model is misspecified, weighting by the inverse of the propensity score may fail to achieve the required covariate balance. One refinement of propensity score weighting is to calibrate the propensity scores by directly incorporating covariate balancing conditions into the construction of the weights (Hainmueller, 2012; Yang, 2018). These calibrated weights $W^{*}_i = W^{*}_i ( T_i, \boldsymbol{X} _i)$ are determined such that they satisfy the following balancing conditions

32 $$\begin{aligned} \sum _{i = 1}^N T_i W^{*}_i \boldsymbol{X}_i = \sum _{i = 1}^N (1-T_i) W^{*}_i \boldsymbol{X}_i = \sum _{i = 1}^N \boldsymbol{X}_i . \end{aligned}$$

In other words, the weights should ensure that the weighted sums of covariates in the treatment and control groups are equal, and that each aligns with the unweighted sum of covariates. The balancing conditions specify which aspects of the weighted covariate distributions should be matched between the treatment and control groups. If interactions between covariates or nonlinear terms (e.g., squared terms) need to be balanced, it is crucial to include these terms in the covariate vector $\boldsymbol{X} _i$ in Eq. (32).

Technically, the calibrated weights $W^{*}_i$ are obtained by refining a set of initial weights $W_i$, defined as the inverse of the estimated propensity scores, that is $W_i = W_i (T_i, \boldsymbol{X}_i ) = T_i/\pi (\boldsymbol{X}_i, \hat{\boldsymbol{\alpha }}) + (1-T_i)/(1-\pi (\boldsymbol{X}_i, \hat{\boldsymbol{\alpha }}))$. More specifically, the calibrated weights $W^{*}_i = W^{*}_i( T_i, \boldsymbol{X} _i, \hat{\boldsymbol{\gamma }}_1, \hat{\boldsymbol{\gamma }}_0)$ are derived using Lagrange multipliers to minimize the Kullback-Leibler (KL) divergence between the calibrated and initial weights, subject to the balancing condition in Eq. (32)

33 $$\begin{aligned} W^{*}_i = \frac{N T_i W_i \exp ( T_i \hat{\boldsymbol{\gamma }}_1^\top \boldsymbol{X}_i)}{\displaystyle \sum _{j = 1}^N T_j W_j \exp ( T_j \hat{\boldsymbol{\gamma }}_1^\top \boldsymbol{X}_j )} + \frac{N (1-T_i) W_i \exp \{ (1-T_i) \hat{\boldsymbol{\gamma }}_0^\top \boldsymbol{X}_i\}}{\displaystyle \sum _{j = 1}^N T_j W_j \exp \{ (1-T_j ) \hat{\boldsymbol{\gamma }}_0^\top \boldsymbol{X}_j \}}, \end{aligned}$$

where $\hat{\boldsymbol{\gamma }}_1$ and $\hat{\boldsymbol{\gamma }}_0$ are vectors of coefficients for the covariates that fulfill the balancing conditions (Fuentes et al., 2022; Yang, 2018). This approach, known as exponential tilting (Chan et al., 2016; Graham et al., 2012), is one of several methods developed to directly optimize covariate balance during estimation of the weights (see Zubizarreta et al., 2023, for a comprehensive overview of related techniques). Simulation studies indicate that calibrated weights can improve performance by increasing stability and covariate balance while reducing weight dispersion, thereby yielding more accurate treatment effect estimates (Chattopadhyay et al., 2020; Deshpande & Kuleshov, 2023; Graham et al., 2012; Imai & Ratkovic, 2014; Zubizarreta, 2015).

The calibrated weights $W^{*}_i$ are used to obtain an estimate of the ATE

34 $$\begin{aligned} \hat{\tau }_\text {CPSW} = \frac{\displaystyle \sum _{i = 1}^N T_i W^{*}_i Y_i}{\displaystyle \sum _{i = 1}^N T_i W^{*}_i} - \frac{\displaystyle \sum _{i = 1}^N (1-T_i) W^{*}_i Y_i}{\displaystyle \sum _{i = 1}^N (1-T_i) W^{*}_i}. \end{aligned}$$

It can be shown that this calibrated propensity score weighting estimator ($\hat{\tau }_\text {CPSW}$) is doubly robust, even though its formulation does not include an explicit outcome model (Fan et al., 2023; Keele et al., 2025; Yang, 2018). In $\hat{\tau }_\text {CPSW}$, the role typically played by an outcome model is instead taken on implicitly by the covariate balancing conditions. We consider the outcome model to be correctly specified when these balancing conditions adequately approximate the true outcome function; equivalently, when the covariates that appear in the true outcome model are included in the covariate balance functions. In this sense, the doubly robust property of $\hat{\tau }_\text {CPSW}$ for the ATE is fulfilled if either (i) the propensity score model is correctly specified, or (ii) the covariate balancing conditions adequately approximate the true outcome function.

First, we show that $\hat{\tau }_\text {CPSW}$ is unbiased if the propensity score model is correctly specified. In this case, the IPW Hajek estimator is unbiased. The inverse propensity score weights serve as initial weights for $\hat{\tau }_\text {CPSW}$. Moreover, when the propensity score model is correctly specified, the balancing conditions in Eq. (32) are already satisfied; that is, the initial weights $W_i = T_i / \pi ( X_i, \hat{\boldsymbol{\alpha }}) + (1-T_i )/ (1-\pi ( X_i, \hat{\boldsymbol{\alpha }}) )$ fulfill

35 $$\begin{aligned} \textsf{E}\big [ W _i T_i \boldsymbol{X}_i \big ] = \textsf{E}\big [ W_i (1-T_i) \boldsymbol{X} _i \big ] = \textsf{E}[ \boldsymbol{X}_i ]. \end{aligned}$$

The identity for the first term can be shown as

36 $$\begin{aligned} \textsf{E}\big [ W _i T_i \boldsymbol{X}_i \big ]= &  \textsf{E}\left[ \textsf{E} \left( \frac{ T_i}{ \pi ( \boldsymbol{X}_i, \hat{\boldsymbol{\alpha }}) } \boldsymbol{X}_i \bigg | \boldsymbol{X}_ i \right) \right] \nonumber \\= &  \textsf{E}\left[ \frac{ \boldsymbol{X}_i}{ \pi ( \boldsymbol{X}_i, \hat{\boldsymbol{\alpha }}) } \textsf{E} \left( T_i | \boldsymbol{X}_ i \right) \right] = \textsf{E} ( \boldsymbol{X}_ i ) . \end{aligned}$$

The second identity in Eq. (35) follows analogously. The weights $W_i^*$ used in $\hat{\tau }_\text {CPSW}$ must also satisfy the balancing constraints and depend on parameters $\hat{\boldsymbol{\gamma }}_1$ and $\hat{\boldsymbol{\gamma }}_0$. Under typical conditions, the solution for determining these weights is unique, which implies that $\hat{\boldsymbol{\gamma }}_1$ and $\hat{\boldsymbol{\gamma }}_0$ are vectors of zeros because the balancing conditions are already satisfied. Consequently, the calibrated weights $W_i^*$ are identical to the initial weights $W_i$ up to a multiplicative factor. Because $\hat{\tau }_\text {CPSW}$ then reduces to the IPW Hajek estimator, the unbiasedness property of the IPW estimator carries over to $\hat{\tau }_\text {CPSW}$.

Second, we show that $\hat{\tau }_\text {CPSW}$ yields asymptotically unbiased estimates when the balancing conditions include all covariates in the outcome model. Assume that the outcome functions are given as $\mu _t(\boldsymbol{X}_i) = \textsf{E}[Y_i \mid \boldsymbol{X} _i, T_i = t ] = \boldsymbol{\beta }_t^\top \boldsymbol{X}_i$ for $t=0,1$. Then the true ATE $\tau$ can be written as

37 $$\begin{aligned} \tau = \textsf{E}[ \mu _1(\boldsymbol{X}_i) - \mu _0(\boldsymbol{X}_i) ] = (\boldsymbol{\beta }_1 - \boldsymbol{\beta }_0)^\top \textsf{E}[ \boldsymbol{X}_i ]. \end{aligned}$$

For the calibrated weights $W_i^*$, the balancing conditions in Eq. (32) imply

38 $$\begin{aligned} \textsf{E} ( W_i^*T_i Y_i )= &  \textsf{E} [ \textsf{E} ( W_i^*T_i Y_i | \boldsymbol{X}_i ) ] = \textsf{E} [ W_i^*T_i \boldsymbol{\beta }_1 ^\top \boldsymbol{X}_i ]\nonumber \\= &  \boldsymbol{\beta }_1 ^\top \textsf{E} [ \boldsymbol{X}_i ] \text { and} \end{aligned}$$

39 $$\begin{aligned} \textsf{E} ( W_i^*(1-T_i) Y_i ) = \boldsymbol{\beta }_0 ^\top \textsf{E} [ \boldsymbol{X}_i ] . \end{aligned}$$

Because $\hat{\tau }_\text {CPSW}$ is based on the difference between the terms in the Equations (38) and (39), it equals the true ATE, and is asymptotically unbiased. Thus, the calibrated propensity score weighting estimator $\hat{\tau }_\text {CPSW}$ provides unbiased estimates of the ATE as long as either the propensity score model used to obtain the initial weights $W_i$ is correctly specified or the balancing conditions include for all relevant covariate terms that are present in the true outcome model, making $\hat{\tau }_\text {CPSW}$ a doubly robust estimator of the ATE.

It is important to note that when covariate balancing is used to estimate the average treatment effect of the treated (ATT), no initial weights from the propensity score model have to be included to ensure the double robustness property (Zhao & Percival, 2017). However, this does not extend to the ATE (Källberg & Waernbaum, 2023).

## Illustrating the effects of misspecification with a single covariate

In this section, we study how model misspecification influences the performance of several ATE estimators in a simplified setting with a single covariate, where *X* follows a standard normal distribution (Waernbaum & Pazzagli, 2023). To keep the subsequent derivations tractable, we specify the data-generating process so that the true outcome model is quadratic in *X* and the true propensity score, $\pi (X) = \mathsf {\textsf {P}}(T = 1 \mid X)$, is also a quadratic function in *X*. Our goal is to specialize the bias formulas from the previous section to this one-covariate setting, thereby characterizing the bias induced by misspecification of the outcome and propensity score models.

Assume that the true propensity score model follows the quadratic form $\pi (X) = a_0 + a_1 X + a_2 X^2$. Since $\textsf{E}(X^2) = 1$, we impose $\textsf{E}[\pi (X)] = 0.5$ by requiring $a_0 + a_2 = 0.5$. Treating the propensity score as misspecified by a linear model, its linear best approximation is then given by $\hat{\pi }(X) = a_0 + a_2 + a_1 X$. The true outcome models are also specified as quadratic functions $\mu _1(X) = b_0 + b_1 X + b_2 X^2$ and $\mu _0(X) = c_0 + c_1 X + c_2 X^2$. The corresponding misspecified linear outcome models are $\hat{\mu }_1(X) = b_0 + b_2 + b_1 X$ and $\hat{\mu }_0(X) = c_0 + c_2 + c_1 X$. In this scenario, the true ATE is given by

40 $$\begin{aligned} \tau = \textsf{E}( \mu _1 (X) - \mu _0 (X) ) = b_0 - c_0 + b_2 - c_2. \end{aligned}$$

The derivations in this section rely on a quadratic Taylor approximation,

41 $$\begin{aligned} \frac{1}{ f_0 + f_1 x + f_2 x^2} \simeq \frac{1}{f_0} - \frac{ f_1}{f_0^2} x + \frac{f_1^2 - f_0 f_2 }{f_0^3} x^2, \end{aligned}$$

which is valid for values of *x* close to zero. This approximation will be used in expressions involving estimated propensity score weights. Assuming that *X* is normally distributed is convenient because all odd moments *X* are zero, which simplifies the resulting expressions.

First, we derive the bias of the estimator obtained from regression estimation (see Eq. (12))

42 $$\begin{aligned} \textsf {Bias}( \hat{\tau }_{\text {Reg}} ) = 2 a_2 (b_2 - c_2). \end{aligned}$$

Thus, bias arises when the true propensity score model includes a quadratic term and the quadratic coefficients in the true outcome models differ between treatment and control. Notably, even under a misspecified linear outcome model, regression estimation yields an unbiased ATE estimate when the quadratic coefficients in $\mu _1(X)$ and $\mu _0(X)$ are equal (i.e., $b_2 = c_2$). Moreover, if the true propensity score model is linear (i.e., $a_2 = 0$), regression estimation is unbiased in this scenario even when the outcome models are misspecified.

Second, we derive the bias of the IPW estimation under model misspecification (see Eq. (16)). Using the Taylor approximation (41), we obtain

43 $$\begin{aligned} \textsf {Bias}( \hat{\tau }_\text {IPW}^\text {HT} ) = 4 a_2 \left[ 4 a_1^2 (b_0 + c_0) + ( 1 + 24 a_1^2 ) (b_2+c_2) - 2 a_1 ( b_1 - c_1 ) \right] . \end{aligned}$$

As expected, Eq. (43) shows that bias occurs whenever the true propensity score model includes a quadratic term (i.e., $a_2 \ne 0$).

Third, we derive the bias of the AIPW estimator under model misspecification (see Eq. (21)) and obtain

44 $$\begin{aligned} \textsf {Bias}( \hat{\tau }_\text {AIPW}^{\text {HT}} ) = 4 a_2 (b_2+c_2) \left( 1 + 20 a_1^2 \right) . \end{aligned}$$

The bias of the AIPW estimator is zero if the propensity score model has no quadratic term ($a_2 \ne 0$) or if both outcome models have no quadratic terms ($b_2 = c_2 = 0$). Interestingly, the bias also vanishes when $b_2 = -c_2$, that is, when the quadratic effects in the outcome models have opposite signs in the treatment and control groups.

Fourth, we derive the bias of the weighted regression estimator (see Eq. (25)). The weighted regression relies on estimated linear regressions $\hat{\mu }_1^W$ and $\hat{\mu }_0^W$ using weights $1/ \hat{\pi }(X)$ and $1/( 1-\hat{\pi }(X))$, respectively. The resulting expressions for the intercept and slope are somewhat cumbersome, but they can be obtained in closed form after applying the Taylor approximation. The linear best approximation of $\hat{\mu }_1^W$ is given by

45 $$\begin{aligned} \hat{\mu }_1^W (X)= &  b_0+\frac{1+12a_1^2+144a_1^4}{1+12a_1^2+48a_1^4} b_2\nonumber \\ &  + \left( b_1-\frac{4a_1}{1+12a_1^2+48a_1^4}b_2 \right) X . \end{aligned}$$

The linear best approximation of $\hat{\mu }_0^W$ is given by

46 $$\begin{aligned} \hat{\mu }_0^W (X)= &  \left( c_0+\frac{1+12a_1^2+144a_1^4}{1+8a_1^2+48a_1^4}\,c_2\right) \nonumber \\ &  +\left( c_1+\frac{4a_1}{1+8a_1^2+48 a_1^4}\,c_2\right) X \end{aligned}$$

Combining these results yields

47 $$\begin{aligned} \textsf {Bias}( \hat{\tau }_{\text {WReg}} ) = 4a_2(b_2+c_2)\frac{1+18a_1^2+168a_1^4+576a_1^6}{1+8a_1^2+48a_1^4}. \end{aligned}$$

As for the AIPW estimator, the bias vanishes if the propensity score model has no quadratic term, or if the quadratic effects in the outcome models have opposite signs so that they cancel each other.

Finally, we compare the bias of the different estimators. The AIPW estimator yields a smaller absolute bias than the estimator obtained from regression estimation if and only if

48 $$\begin{aligned} (1 + 20 a_1^2) |b_2 + c_2| < \frac{1}{2} |b_2 -c_2| . \end{aligned}$$

Thus, in this scenario with quadratic effects, AIPW can be expected to be doubly robust only when the quadratic coefficients $b_2$ and $c_2$ in the outcome models have opposite signs. Consequently, in this setting, it may be difficult for the AIPW estimator to outperform regression estimation with respect to bias. Moreover, the derivations show that the absolute bias of the weighted regression estimator (see Eq. (47) is always less than or equal to that of the AIPW estimator (see Eq. (44), which is a notable finding. However, it is important to emphasize that this ordering is specific to the illustrative setting considered here.

## Simulation studies

In the previous paragraphs, we discussed four doubly robust approaches designed to mitigate misspecification bias, which arises from using an incorrect model for statistical adjustment. These estimators combine outcome modeling with a treatment model, ensuring unbiased estimates of the ATE as long as at least one of the two models is correctly specified. Importantly, the researcher does not need to know which model is correct, reducing sensitivity to potential misspecifications that may occur when relying on a single analysis model for statistical adjustment.

In the following, we present the results of two simulation studies comparing the performance of the four doubly robust methods with the two traditional approaches that rely on a single model (regression estimation and IPW). In the first study, we evaluate the estimators under four data-generating scenarios: (a) both the outcome and propensity score models are correctly specified, (b) only the propensity score model is correct, (c) only the outcome model is correct, and (d) both models are misspecified. Our primary focus is on assessing the performance differences among the doubly robust methods in the first three scenarios, where at least one model is correctly specified. In the fourth scenario, where both models are misspecified, it is difficult to predict which method performs best, as their effectiveness strongly depends on the data-generating mechanism and the nature of the misspecifications. Prior research, as well as our single-covariate illustration, suggests that when both nuisance models are misspecified, doubly robust estimators may produce “doubly fragile” ATE estimates that are not necessarily superior to those obtained using a single-model approach (Cao et al., 2009; Ding, 2024; Imai & Ratkovic, 2014; Kang & Schafer, 2007; Robins et al., 2007; Vermeulen & Vansteelandt, 2015). In addition, we examine three key practical considerations in specifying doubly robust methods. First, we assess whether truncating extreme propensity score weights improves estimator performance (Cole & Hernán, 2008; Petersen et al., 2012). Second, we compare the AIPW and IPW estimators using two types of weights: HT weights and the normalized Hajek weights (Khan & Ugander, 2023; Lunceford & Davidian, 2004; Millimet & Tchernis, 2009). Third, we investigate potential efficiency losses due to overly complex model specifications, such as incorporating quadratic effects when the true data-generating process only involves linear covariate effects (Dehejia & Wahba, 2002; Millimet & Tchernis, 2009).

In the second simulation study, we adopted the data-generating mechanism from the simulation study of Kang and Schafer (2007) and examined whether our conclusions about the performance of the four doubly robust methods could be generalized to a different data-generating process.

### Simulation Study 1

In Simulation Study 1, we evaluated the performance of various estimators across four data-generating mechanisms (DGMs). In DGM1, both the outcome regression (OR) model and the propensity score (PS) model were linear in the covariates. In DGM2, the OR model was exponential while the PS model remained linear. In DGM3, the OR model was linear, whereas the PS model was exponential. Finally, in DGM4, both the OR and PS models were exponential. We used the exponential function as a monotone nonlinear functional misspecification as in Ding (2024). Whenever the OR or PS model was exponential, applying a linear analysis model resulted in a misspecification of the functional relationship—either between the outcome and the covariates or between the treatment and the covariates (for an illustration of misspecification with exponential models, see Figure 1 in Supplement A). We manipulated the sample size, the proportion of treated individuals, the proportion of variance explained by the covariates in both the outcome and treatment models, and the number of covariates.

#### Method

For the DGMs, we simulated random variables $\boldsymbol{X}$, *T* and *Y* with realizations $\boldsymbol{x} _i$, $t_i$, and $y_i$ for $i = 1, \dots , N$. Each covariate $X_k$ ($k = 1,\dots ,K$) was independently sampled from a standard normal distribution $\text {N}(0, 1)$ and truncated to the range $[-3, 3]$ to prevent extreme values. All covariates are assumed to be uncorrelated, and the number of covariates *K* was set to either 2 or 6.

In DGM1, the OR and PS models were linear, meaning that the covariates $X_k$ had only linear effects in both models. The linear PS model was defined as

49 $$\begin{aligned} \pi _i = {\text {expit}}\left( \alpha _0 + \sum _{k = 1}^K \alpha _k x_{ik}\right) . \end{aligned}$$

For $K=2$ covariates, the vector of coefficients was set to $\boldsymbol{\alpha } = \omega (1, 1)^\top$, and for $K=6$ it was set to $\boldsymbol{\alpha } = \omega (1, 1, 1, 1, 1, 1)^\top$. The scaling factor $\omega$ was adjusted to achieve the desired $R^2$ for the PS model (0.25 or 0.50). The $R^2$ was computed as $v_\pi / (v_\pi + 3.29)$, where $v_\pi = \text {Var} \left( \sum _{k = 1}^K \alpha _k x_{ik} \right)$ is the variance of the logit-transformed propensity scores and 3.29 is the variance of the logistic distribution. Treatment assignment $t_i$ is determined from a Bernoulli distribution with probability $\pi _i$, such that $t_i \sim \text {Bernoulli}(\pi _i)$. The intercept $\alpha _0$ was adjusted to ensure the desired treatment proportion of either 0.30 or 0.50.

The linear OR model was defined as

50 $$\begin{aligned} y_i = t_i \sum _{k = 1}^K \beta _{1k} x_{ik} + (1 - t_i) \sum _{k = 1}^K \beta _{0k} x_{ik} + \varepsilon _{i}, \end{aligned}$$

where the residuals $$\varepsilon _i$$ were independently drawn from a normal distribution $$\text {N}(0, \sigma ^2)$$. For $$K=2$$ covariates, the coefficient vectors were set as $$\boldsymbol{\beta }_1 = w_1(2, 1)^\top $$ and $$\boldsymbol{\beta }_0 = w_0(-2, 1)^\top $$. For $$K=6$$ covariates, they were defined as $$\boldsymbol{\beta }_1 = w_1(2, 1, 2, 1, 2, 1)^\top $$ and $$\boldsymbol{\beta }_0 = w_0(-2, 1, -2, 1, -2,$$$$1)^\top $$. The scaling factors $$w_1$$, $$w_0$$, along with the residual variance $$\sigma ^2$$ were adjusted to ensure that the variance of the outcome was equal to 1, and that the OR model achieved the desired $$R^2$$ values of 0.25 or 0.50. The values of $$R^2$$ are defined based on the true data models and measure the strength of systematic covariate information relative to noise. Fixing it across functional forms ensures comparable estimation difficulty while magnifying the impact of correct versus misspecified models.

In DGM2, the propensity scores were generated from a model with only linear effects of the covariates as in Eq. (49). However, in the OR model, the covariates $X_k$ entered the regression through an exponential transformation

51 $$\begin{aligned} y_i = t_i \sum _{k = 1}^K \beta _{1k} \exp (x_{ik}) + (1-t_i)\sum _{k = 1}^K \beta _{0k} \exp (x_{ik}) + \varepsilon _{i} . \end{aligned}$$

For $K=2$ covariates, the coefficient vectors were set as $\boldsymbol{\beta }_1 = w_1(2,1)^\top$ and $\boldsymbol{\beta }_0 = w_0(-2,-1)^\top$. For $K=6$ covariates, they were defined as $\boldsymbol{\beta }_1 = w_1(2,1,2,1,2,1)^\top$ and $\boldsymbol{\beta }_0 = w_0(-2,-1,-2,-1, -2,-1)^\top$.

In DGM3, the OR model was linear (see Eq. (50)), while the PS model followed an exponential form

52 $$\begin{aligned} \pi _i = {\text {expit}}\left( \alpha _0 + \sum _{k = 1}^K \alpha _k \exp (x_{ik})\right) , \end{aligned}$$

where the coefficient vector was set to $\boldsymbol{\alpha } = \omega (1,-1)^\top$ for $K=2$ covariates, while $\boldsymbol{\alpha } = \omega (1,-1,1,-1,1,-1)^\top$ for $K=6$ covariates. Finally, in DGM4 both the OR and PS models followed an exponential form. The OR model was defined as in Eq. (51) from DGM2, and the PS model followed Equation (52) from DGM3.

The true ATE depends on the OR model coefficients and the covariate distribution

53 $$\begin{aligned} \tau = ( \boldsymbol{\beta }_1 - \boldsymbol{\beta }_0 )^\top \textsf{E}( \boldsymbol{X} ) = 0 \text { or } \tau = ( \boldsymbol{\beta }_1 - \boldsymbol{\beta }_0 )^\top \textsf{E}( \exp ( \boldsymbol{X} ) ) \ne 0, \end{aligned}$$

where $\exp ( \boldsymbol{X} )$ represents the exponential transformation for each covariate, that is $\exp ( \boldsymbol{X}) = ( \exp (X_1), \dots , \exp (X_K))$. In DGM1 and DGM3, where the OR model includes only linear terms in $\boldsymbol{X}$, the true ATE is zero. However, in DGM2 and DGM4, where $\boldsymbol{X}$ appears in the OR model through an exponential transformation, the true ATE is nonzero. Note that the intercepts in the OR models were set to zero.

In our simulation design, we varied the $R^2$ value, sample size (*N*), and treatment proportion. The $R^2$ values were kept equal for both the OR and PS models and were set to either 0.25 or 0.50. Sample sizes were selected to evaluate both finite-sample performance and asymptotic properties. For finite-sample evaluation, we considered *N* values of 500, 1000, and 5000. To approximate the asymptotic bias, we used a substantially larger sample size of $N = 2 \times 10^6$. Treatment proportions ($p_T$) were set at 0.30 and 0.50, representing unbalanced and balanced treatment assignments, respectively. For each DGM and number of covariates (*K* = 2 or 6), this resulted in a total of 2 ($R^2$) $\times$ 4 (*N*) $\times$ 2 ($p_T$) $= 16$ simulation conditions. For finite-sample analyses (*N* = 500, 1000, and 5000), we generated 1000 replications per condition, while for asymptotic analyses ($N = 2 \times 10^6$) we conducted 50 replications per condition.

For each simulated replication, we specified analysis models for both OR and PS models. In the OR model, we used OLS to regress the outcome variable *Y* on the covariates separately for the treated and control groups. This produced predicted values $\mu _1(\boldsymbol{x} _i, \hat{\boldsymbol{\beta }}_1)$ and $\mu _0(\boldsymbol{x} _i, \hat{\boldsymbol{\beta }}_0)$, which were used to obtain an estimate of the ATE (see Eq. (11)). We also investigated two levels of model complexity. The linear specification included only the first-order covariate terms, $\{X_{1}, \dots , X_{K}\}$, while the nonlinear model incorporated quadratic terms and interactions, $\{X_{1}, \dots , X_{K}, X_{1}^2, \dots , X_{K}^2, X_{1}X_{2}, \dots , X_{(K-1)}X_{K}\}$. The linear specification was used as the default for calculating the regression estimator (Reg), and the effect of a more complex model specification was explored in additional simulations.

For the PS model, logistic regression was used to regress the treatment variable *T* on the covariates, yielding the propensity scores $\pi (\boldsymbol{x} _i, \hat{\boldsymbol{\alpha }})$. After estimating the propensity scores, we applied truncation at three levels (see Eq. (18)): $\text {TR0} = [0.0001, 0.9999]$, $\text {TR1} = [0.01, 0.99]$, and $\text {TR5}=[0.05, 0.95]$. As with the OR model, the PS model could be specified as either linear or quadratic. To estimate the ATE, we used the IPW estimator with normalized (Hajek) weights, applying truncation at TR1 and using a linear PS model as the default specification ($\hat{\tau }_\text {IPW}^\text {Hajek}$; see Eq. (17)), which is reported in the main results. Additional simulations examined the effects of weight normalization (HT vs. Hajek weights), the truncation levels (TR0, TR1, or TR5), and increased PS model complexity (linear vs. nonlinear) on the IPW estimator.

**Table 1:** $R^2$ values of linear nuisance models under misspecification in Simulation Study 1

		$R^2$ of misspecified model			
True model		#Cov = 2		#Cov = 6	
$p_T$	$R^2$	OR	PS	OR	PS
0.5	0.25	0.16	0.14	0.17	0.15
	0.50	0.33	0.27	0.33	0.30
0.3	0.25	0.16	0.15	0.17	0.15
	0.50	0.33	0.29	0.33	0.32

*Note.* #Cov = number of covariates; $p_T$ = treatment proportion; OR = outcome regression; PS = propensity score. True models are exponential, while $R^2$ values for the misspecified nuisance models are computed using linear models with a large sample ($N = 2\times 10^6$)

To provide a quantitative sense of the degree of model misspecification in Simulation Study 1, Table 1 reports the $R^2$ values of misspecified linear models under settings where the true data-generating models are exponential. For the OR model, the linear misspecified model attains $R^2$ values between 0.16 and 0.17 when the true model has $R^2 = 0.25$, and $R^2 = 0.33$ when the true model has $R^2 = 0.50$. For the PS model, the linear misspecified model achieves $R^2$ values between 0.14 and 0.15 when the true model has $R^2 = 0.25$, and between 0.27 and 0.32 when the true model has $R^2 = 0.50$. Overall, the misspecification design in Simulation Study 1 induces a moderate reduction in explained variance relative to the true models, which indicates a moderate amount of misspecification that is more pronounced in the conditions with $R^2=0.50$.

The AIPW estimator extends regression-based estimation by augmenting the outcome model with inverse-propensity-weighted residuals. For the main simulation results, we used the AIPW estimator with Hajek weights ($\hat{\tau }_\text {AIPW}^\text {Hajek}$), applying truncation at TR1 and specifying both the OR and PS models as linear. Additional simulations explored the impact of weight normalization, the truncation levels, and model complexity of the OR and PS models.

For regression weighted by the inverse of the propensity score (WReg), we employed WLS to estimate the regression coefficients for the outcome models, using inverse propensity scores as weights (see Eq. (23)). For the main results, we applied truncation at TR1 and specified both the OR and PS models as linear. Note that the WReg estimator does not require a choice between HT or Hajek weights, as the regression residuals are inherently normalized.

Regression with the inverse of the propensity score as a clever covariate (Clev) incorporates the inverse of the propensity score as an additional covariate in a two-stage procedure. The PS model was estimated for constructing the clever covariate (see Eq. (26)). For the main simulation, we specified linear OR and PS models, and truncated the propensity score at TR1.

Finally, for the calibrated propensity score weighting estimator (CPSW), balancing weights were generated by calibrating the initial weights to satisfy covariate balancing conditions (see Eq. (33)). Similar to the OR model, these conditions targeted either first moments (linear specification) or included first and second moments along with interactions (quadratic specification). Analogous to the PS model, the initial weights could be a constant vector (all elements equal to 1) or propensity scores obtained from a PS model with a linear or quadratic specification. The propensity scores used to compute the initial weights were truncated at different levels prior to calibration (TR0, TR1, or TR5). Additionally, balancing weights were bounded based on PS truncation levels: for TR0, the maximum is $10^9$; for TR1, it is $1/0.01 = 100$; and for TR5, $1/0.05 = 20$. For the main simulation results, we used a linear specification for the PS model and the balancing conditions, and truncated propensity scores at TR1, and bounded the balancing weights at 100.

**Table 2:**
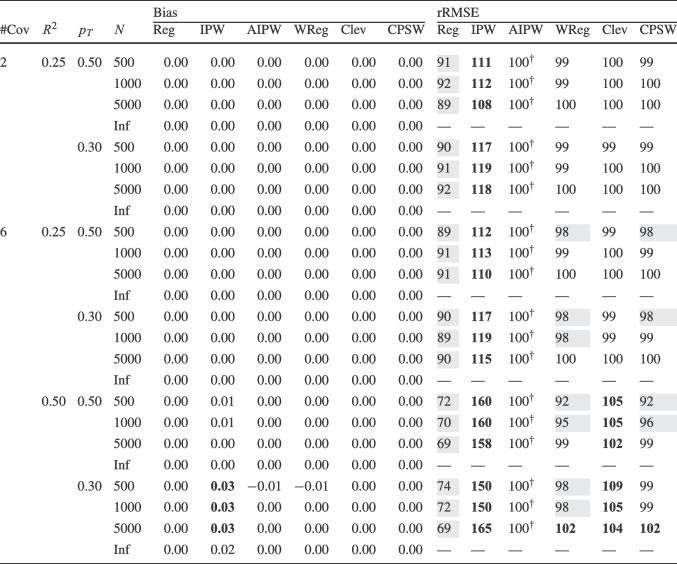
Simulation Study 1: Bias and relative root mean square error (rRMSE) of estimators as a function of the number of covariates (#Cov), $R^2$, treatment proportion ($p_T$), and sample size (*N*) under data-generating model 1, where the true outcome regression model is linear, and the true propensity score model is linear

*Note*. Inf = infinite, computed with large samples ($N = 2 \times 10^6$); Reg = regression estimation; IPW = inverse probability weighting; AIPW = augmented inverse probability weighting; WReg = regressions weighted by the inverse of the propensity score; Clev = regression with the inverse of the propensity score as a clever covariate; CPSW= calibrated propensity score weighting. The $R^2$ value applies to both the true OR and true PS models. In analysis models, propensity scores are truncated at [0.01, 0.99], with linear specifications for both OR and PS. For IPW and AIPW, weights are normalized (Hajek type). The rRMSE values are ratios multiplied by 100 for clarity, with AIPW as the reference ($100^\dag$) in each data constellation (#Cov $\times$ $R^2$ $\times$ $p_T$ $\times$ *N*). Absolute bias values ($|\text {Bias}|$) $\ge 0.03$ and rRMSE values $\ge 102$ are bolded, while rRMSE values $\le 98$ are shaded in grey

We evaluated the different estimators of the ATE using two criteria: bias and root-mean-square error (RMSE). Bias was calculated as the raw deviation between the estimated treatment effect and the true treatment effect within each design cell. To mitigate the influence of extreme outliers in ATE estimates, all estimates were truncated within the range $[-3, 3]$. Since outcome variables were standardized, we considered a bias magnitude of 0.03 or less acceptable, referring to such estimators as approximately unbiased. Overall accuracy was assessed using empirical RMSE, which combines the squared bias and the variance of parameter estimates into a single measure of precision. To facilitate performance comparisons across estimators, we computed the relative RMSE (rRMSE) as a percentage. This was calculated by dividing the RMSE of the estimator of interest by that of the reference estimator (i.e., AIPW with Hajek weights, PS truncated at TR1, and linear OR and PS models) then multiplying by 100 for interpretability. An rRMSE of 102 or higher indicated a significant efficiency loss compared to the reference estimator, whereas an rRMSE of 98 or lower represented a significant efficiency gain. We used AIPW as the reference estimator because it can be considered the prototypical doubly robust estimator of the ATE (Ding, 2024; Glynn & Quinn, 2010; Kurz, 2022; Lunceford & Davidian, 2004). All analyses for the simulation study were carried out with the statistical software R (Version 4.3.1; R Core Team, 2023). The OR model was estimated with the stats::lm() function from the R package stats. The PS model was estimated with the arm::bayesglm() function from the R package arm (Version 1.14-4; Gelman & Su, 2024). We used bayesglm() rather than stats::glm() to reduce instability due to (quasi-)complete separation, which can occur in small samples and lead to extreme coefficient estimates. To mitigate this issue, we specified Student’s *t* prior distributions for the regression coefficients with a scale of 20 and 4 degrees of freedom (Gelman et al., 2008). The codes for reproduction of results and online supplementary material are available at https://osf.io/6veaj/.

**Table 3:**
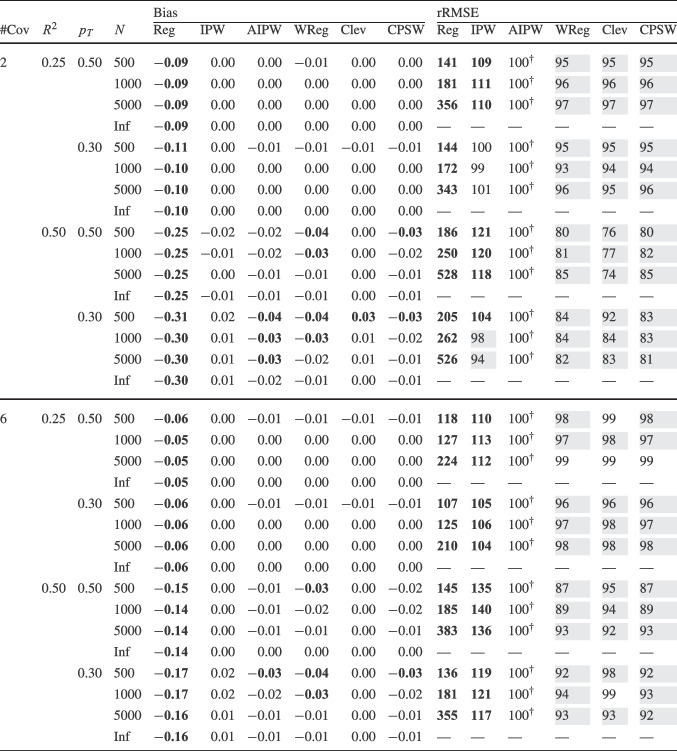
Simulation Study 1: Bias and relative root mean square error (rRMSE) of estimators as a function of the number of covariates (#Cov), $R^2$, treatment proportion ($p_T$), and sample size (*N*) under data-generating model 2, where the true outcome regression model is exponential, and the true propensity score model is linear

*Note*. Inf = infinite, computed with large samples ($N = 2 \times 10^6$); Reg = regression estimation; IPW = inverse probability weighting; AIPW = augmented inverse probability weighting; WReg = regressions weighted by the inverse of the propensity score; Clev = regression with the inverse of the propensity score as a clever covariate; CPSW= calibrated propensity score weighting. The $R^2$ value applies to both the true OR and true PS models. In analysis models, propensity scores are truncated at [0.01, 0.99], with linear specifications for both OR and PS. For IPW and AIPW, weights are normalized (Hajek type). The rRMSE values are ratios multiplied by 100 for clarity, with AIPW as the reference ($100^\dag$) in each data constellation (#Cov $\times$ $R^2$ $\times$ $p_T$ $\times$ *N*). Absolute bias values ($|\text {Bias}|$) $\ge 0.03$ and rRMSE values $\ge 102$ are bolded, while rRMSE values $\le 98$ are shaded in grey

#### Results

Table 2 summarizes the bias and rRMSE of the six estimators under DGM1, where the true OR and PS models were both linear, ensuring correct specification when linear analysis models were used. In terms of bias, nearly all estimators produced unbiased ATE estimates (close to 0.00), except for IPW, which exhibited biased estimates under certain conditions. Specifically, when $R^2 = 0.5$ and $p_T = 0.3$, the bias for IPW ranged from 0.02 to 0.05. Regarding RMSE, Reg achieved the lowest rRMSE values (68 to 92), indicating the highest estimation stability. While the doubly robust estimators were less efficient than Reg, they consistently outperformed IPW (doubly robust: 92 to 109; IPW: 108 to 166). Among doubly robust estimators, WReg and CPSW performed best in smaller samples ($N = 500$ or 1000), though performance differences between doubly robust estimators diminished in larger samples. However, Clev showed some efficiency loss when $R^2 = 0.5$ (Clev: 102 to 109). Overall, larger $R^2$ values (e.g., $R^2 = 0.5$) amplified the efficiency differences between the estimators. However, the number of covariates ($K = 2$ or $K = 6$) did not affect the relative performance of the estimators.

Table 3 presents the results for DGM2, where the true OR model followed an exponential form, and the true PS model was linear. With linear analysis models, the OR model was misspecified, whereas the PS model remained correct. This misspecification led Reg to produce biased and less precise estimates (Bias: $-0.31$ to $-0.05$; rRMSE: 107 to 528). In contrast, doubly robust estimators were nearly unbiased and more accurate (Bias: $-0.04$ to 0.00; rRMSE: 74 to 100). In small samples (e.g., $N = 500$ or 1000) with $R^2 = 0.5$, doubly robust estimators exhibited slight bias (nearly between $-0.04$ and $-0.02$), though still substantially lower than that of Reg. Among doubly robust methods, WReg, CPSW, and Clev consistently produced more precise estimates than AIPW (WReg: 80 to 98; Clev: 74 to 99; CPSW: 80 to 99). However, these differences in rRMSE between the doubly robust estimators were less pronounced with a larger number of covariates. Notably, IPW was generally less precise than AIPW (IPW: 94 to 140)^2^, reinforcing the benefit of augmenting IPW with an outcome model to improve efficiency.

Table 4 presents the results for DGM3, where the true OR model was linear, and the true PS model was exponential. With linear analysis models, the OR model was correct, while the PS model was misspecified. Because Reg and doubly robust estimators relied on the correctly specified OR model, they produced unbiased estimates (Reg and doubly robust: 0.00). In contrast, IPW estimates were generally biased due to the misspecified PS model (IPW: $-0.23$ to $-0.02$). Regarding rRMSE, Reg achieved the lowest values (82 to 99), demonstrating the highest precision, consistent with its performance in DGM1. Doubly robust estimators followed closely, exhibiting only minor efficiency losses (doubly robust: 93 to 100). In contrast, IPW showed substantially higher rRMSE values (122 to 893), highlighting the impact of PS model misspecification. Among doubly robust estimators, WReg, CPSW, and Clev performed similarly to AIPW in most conditions but occasionally outperformed AIPW, particularly when $R^2 = 0.5$ and $p_T = 0.3$.

Table 5 summarizes the results for DGM4, where both the OR and PS models were exponential, leading to misspecification under linear analysis models. As expected, all estimators exhibited some degree of bias. However, the “doubly fragile” phenomenon, as discussed in Kang and Schafer (2007) and Ding (2024, p. 157), did not occur. Instead, doubly robust estimators had smaller biases compared to single-model estimators (doubly robust: $-0.05$ to 0.05; single model: $-0.18$ to $-0.02$), likely due to error cancellation between the two misspecified models. This was also reflected in the rRMSE, where doubly robust estimators provided more accurate estimates than single-model estimators (doubly robust: 45 to 123; single model: 99 to 389). Among doubly robust estimators, WReg, CPSW, and Clev outperformed AIPW, especially when treatment assignment was unbalanced ($p_T = 0.3$). However, performance differences among doubly robust estimators diminished when the number of covariates increased.

**Table 4:**
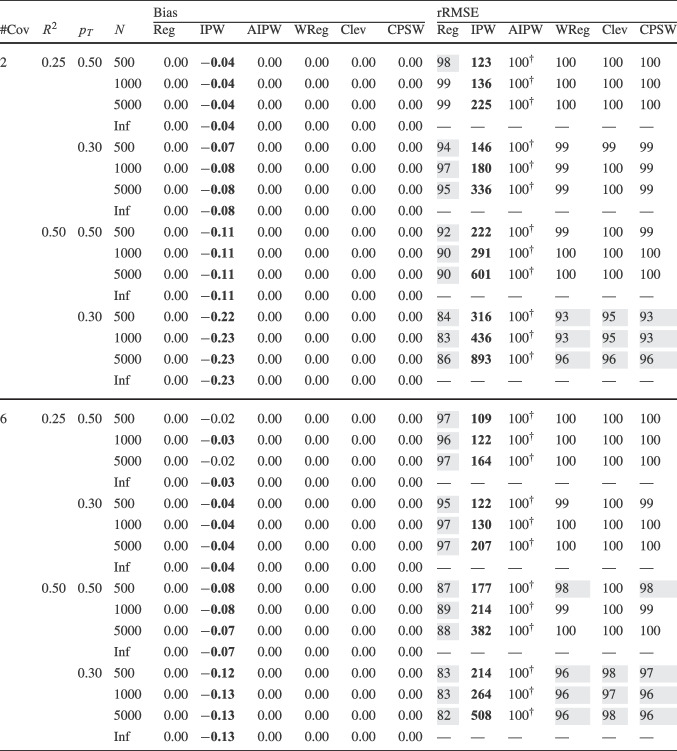
Simulation Study 1: Bias and relative root-mean-square error (rRMSE) of estimators as a function of the number of covariates (#Cov), $R^2$, treatment proportion ($p_T$), and sample size (*N*) under data-generating model 3, where the true outcome regression model is linear, and the true propensity score model is exponential

*Note*. Inf = infinite, computed with large samples ($N = 2 \times 10^6$); Reg = regression estimation; IPW = inverse probability weighting; AIPW = augmented inverse probability weighting; WReg = regressions weighted by the inverse of the propensity score; Clev = regression with the inverse of the propensity score as a clever covariate; CPSW= calibrated propensity score weighting. The $R^2$ value applies to both the true OR and true PS models. In analysis models, propensity scores are truncated at [0.01, 0.99], with linear specifications for both OR and PS. For IPW and AIPW, weights are normalized (Hajek type). The rRMSE values are ratios multiplied by 100 for clarity, with AIPW as the reference ($100^\dag$) in each data constellation (#Cov $\times$ $R^2$ $\times$ $p_T$ $\times$ *N*). Absolute bias values ($|\text {Bias}|$) $\ge 0.03$ and rRMSE values $\ge 102$ are bolded, while rRMSE values $\le 98$ are shaded in grey

**Table 5:**
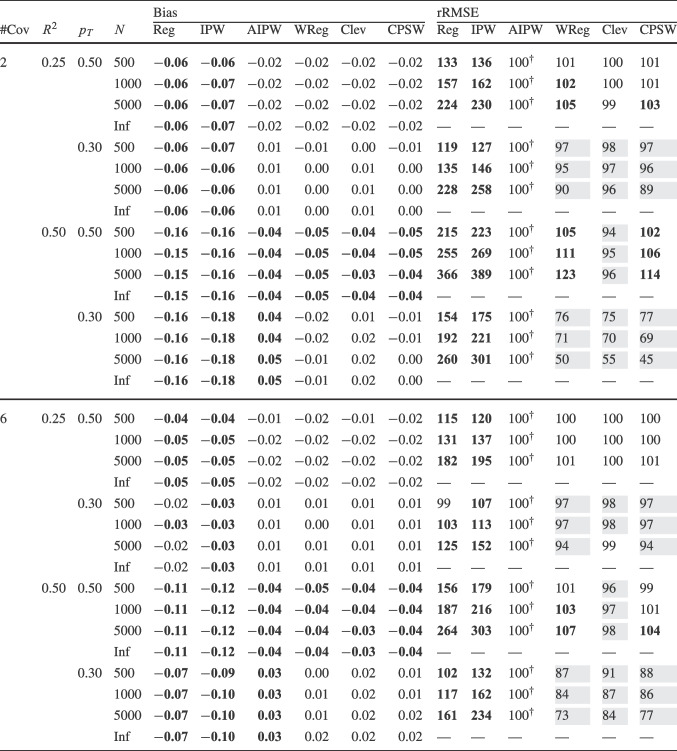
Simulation Study 1: Bias and relative root-mean-square error (rRMSE) of estimators as a function of the number of covariates (#Cov), $R^2$, treatment proportion ($p_T$), and sample size (*N*) under data-generating model 4, where the true outcome regression model is exponential, and the true propensity score model is exponential

*Note*. Inf = infinite, computed with large samples ($N = 2 \times 10^6$); Reg = regression estimation; IPW = inverse probability weighting; AIPW = augmented inverse probability weighting; WReg = regressions weighted by the inverse of the propensity score; Clev = regression with the inverse of the propensity score as a clever covariate; CPSW= calibrated propensity score weighting. The $R^2$ value applies to both the true OR and true PS models. In analysis models, propensity scores are truncated at [0.01, 0.99], with linear specifications for both OR and PS. For IPW and AIPW, weights are normalized (Hajek type). The rRMSE values are ratios multiplied by 100 for clarity, with AIPW as the reference ($100^\dag$) in each data constellation (#Cov $\times$ $R^2$ $\times$ $p_T$ $\times$ *N*). Absolute bias values ($|\text {Bias}|$) $\ge 0.03$ and rRMSE values $\ge 102$ are bolded, while rRMSE values $\le 98$ are shaded in grey

Truncating extreme propensity scores can help stabilize estimators that rely on propensity scores, but it may also introduce bias, creating a trade-off between variance reduction and bias. Table 6 presents the bias and rRMSE of the six estimators at three truncation levels (TR0, TR1, and TR5) across four DGMs with two covariates. As shown in the Table 6, TR1 consistently improved estimator performance when the PS model was correctly specified and never led to efficiency loss in any scenario within our simulations. TR5 also enhanced performance in many cases, particularly for doubly robust estimators in DGM1, in small samples under DGM2 (e.g., $N = 500$), and when solely the PS model was misspecified (DGM3). However, TR5 introduced asymptotic bias under certain conditions, notably for IPW under DGM1 and for both IPW and doubly robust estimators under DGM2. Notably, the linear PS analysis model was correctly specified in DGM1 and DGM2. This suggests that under TR5, the propensity scores no longer corresponded to the correctly specified PS model, leading to inadequate control for covariates. As a result, this bias contributed to higher rRMSE values at larger sample sizes (e.g., $N = 5000$). Overall, a similar pattern of results was observed in conditions with six covariates (see Table S4 to Table S7 in Supplement A).

**Table 6:**
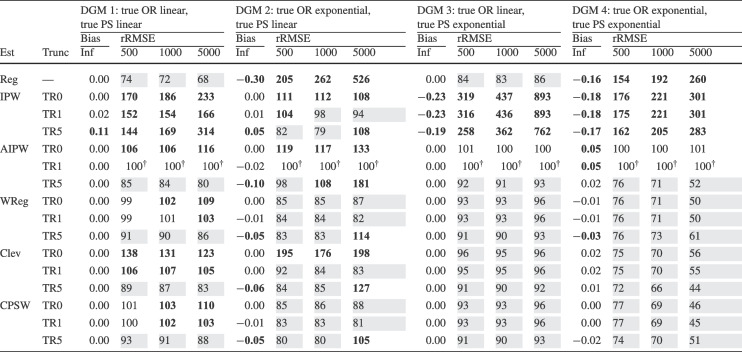
Simulation Study 1: Bias and relative root-mean-square error (rRMSE) of estimators as a function of propensity score truncation levels (Trunc) across different data-generating models (DGM) and sample sizes, for data with two covariates, $$ R^2 = {0.50} $$, and treatment proportion $$ = {0.30}$$

*Note*. OR = outcome regression; PS = propensity score; Est = estimator; Trunc = propensity score truncation level; Inf = infinite, computed with large samples ($N = 2 \times 10^6$); Reg = regression estimation; IPW = inverse probability weighting; AIPW = augmented inverse probability weighting; WReg = regressions weighted by the inverse of the propensity score; Clev = regression with the inverse of the propensity score as a clever covariate; CPSW= calibrated propensity score weighting; TR0 = [0.0001, 0.9999]; TR1 = [0.01, 0.99]; TR5 = [0.05, 0.95]. The $R^2$ value applies to both the true OR and true PS models. In analysis models, specifications for both OR and PS are linear. For IPW and AIPW, weights are normalized (Hajek type). The rRMSE values are ratios multiplied by 100 for clarity, with AIPW TR1 as the reference ($100^\dag$) in each data constellation (DGM $\times$ *N*). Absolute bias values ($|\text {Bias}|$) $\ge 0.03$ and rRMSE values $\ge 102$ are bolded, while rRMSE values $\le 98$ are shaded in grey

**Table 7:**
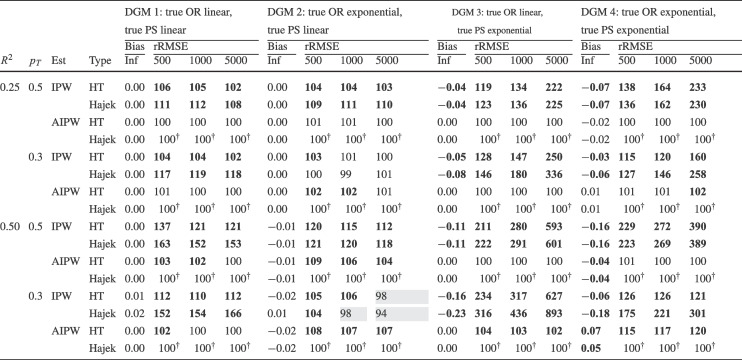
Simulation Study 1: Bias and relative root mean square error (rRMSE) of estimators as a function of $R^2$, treatment proportion ($p_T$), and weight type across different data-generating models (DGM) and sample sizes, for data with two covariates

*Note*. OR = outcome regression; PS = propensity score; Est = estimator; Inf = infinite, computed with large samples ($N = 2 \times 10^6$); IPW = inverse probability weighting; AIPW = augmented inverse probability weighting. The $R^2$ value applies to both the true OR and true PS models. In analysis models, propensity scores are truncated at [0.01, 0.99], with linear specifications for both OR and PS. The rRMSE values are ratios multiplied by 100 for clarity, with AIPW Hajek as the reference ($100^\dag$) in each data constellation ($R^2$ $\times$ $p_T$ $\times$ DGM $\times$ *N*). Absolute bias values ($|\text {Bias}|$) $\ge 0.03$ and rRMSE values $\ge 102$ are bolded, while rRMSE values $\le 98$ are shaded in grey

**Table 8:**
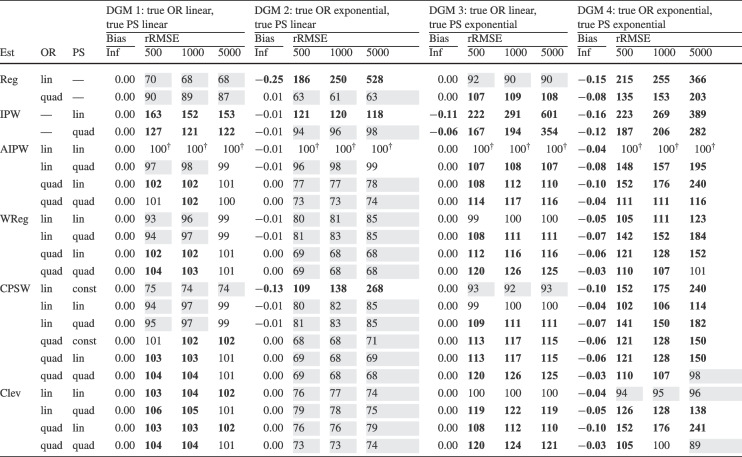
Simulation Study 1: Bias and relative root-mean-square error (rRMSE) of estimators as a function of analysis model specifications for outcome regression (OR) and propensity score (PS) across different data-generating models (DGM) and sample sizes, for data with two covariates, $$ R^2 = 0.50$$, and treatment proportion $$ = {0.5}$$

*Note*. Est = estimator; Inf = infinite, computed with large samples ($N = 2 \times 10^6$); Reg = regression estimation; IPW = inverse probability weighting; AIPW = augmented inverse probability weighting; WReg = regressions weighted by the inverse of the propensity score; Clev = regression with the inverse of the propensity score as a clever covariate; CPSW= calibrated propensity score weighting; lin = linear specification for analysis model; quad = quadratic specification (with interaction term) for analysis model; const = constant 1 as initial weights. The $R^2$ value applies to both the true OR and true PS models. In analysis models, propensity scores are truncated at [0.01, 0.99]. For IPW and AIPW, weights are normalized (Hajek type). The rRMSE values are ratios multiplied by 100 for clarity, with AIPW lin OR $\times$ lin PS as the reference ($100^\dag$) in each data constellation (DGM $\times$ *N*). Absolute bias values ($|\text {Bias}|$) $\ge 0.03$ and rRMSE values $\ge 102$ are bolded, while rRMSE values $\le 98$ are shaded in grey

Table 7 compares HT and Hajek weights for the IPW and AIPW estimators at a moderate truncation level (TR1) under conditions with two covariates. For AIPW, both types of weights led to similar performance. However, Hajek weights were consistently preferred, as they never led to efficiency loss and always performed as well as or better than HT weights. However, for IPW, Hajek weights generally increased bias and reduced efficiency. They only improved performance of the IPW estimator in a few cases, such as under certain conditions with six covariates (see Table S8 in Supplement A) or when treatment assignment was unbalanced ($p_T = 0.3$) in DGM2, where the PS model was correctly specified.

Table 8 compares the performance of the six estimators under different specifications of the nuisance models, considering both linear and quadratic specifications for the OR and PS models. Our focus is on conditions where treatment assignment was balanced ($p_T = 0.5$), the coefficient of determination was $R^2 = 0.50$, and propensity scores were truncated at TR1 (see Tables S9 to S15 in Supplement A for additional conditions). For Reg and IPW, using quadratic specifications for their respective nuisance models improved precision when the true models followed an exponential form by reducing bias. However, when the true OR model was linear, specifying a quadratic OR model led to efficiency losses in Reg due to overfitting. Interestingly, despite this overfitting, Reg often still outperformed AIPW in terms of rRMSE. Another notable finding is that IPW with a quadratic PS model yielded efficiency gains even when the true PS model had only linear effects. For doubly robust estimators, incorporating quadratic OR models had a similar effect as in Reg: It improved precision when the true OR model followed an exponential form, but led to efficiency losses when the true OR model was linear. Nevertheless, specifying a quadratic PS model generally did not provide an advantage for doubly robust estimators. When the true PS model was linear, only AIPW benefited from a quadratic PS specification. However, when the true PS model was exponential, this specification resulted in overall efficiency losses. For CPSW, constant initial weights performed best in settings with a linear OR model but only when the true OR model was also linear. Under these conditions, CPSW performed similarly to Reg, particularly when the balancing conditions for calibrating the PS scores included only the first moments of the covariates. However, when the true OR model followed an exponential form, using constant initial weights introduced asymptotic bias. This bias was mitigated by incorporating second moments and interaction terms into the balancing conditions for the covariates.

#### Summary

The main results of Simulation Study 1 can be summarized as follows. First, when both models were correctly specified (DGM1), all estimators were unbiased. Reg had the lowest RMSE and performed best, followed by doubly robust estimators, which showed a slight loss of efficiency. IPW had the lowest precision. Second, with partial misspecification (DGM2 and DGM3), single-model estimators became biased and less precise, whereas doubly robust estimators remained unbiased and accurate. Third, under full misspecification (DGM4), doubly robust estimators outperformed single-model estimators, likely due to error cancellation between the two misspecified models. Thus, we found no evidence that doubly robust estimators were “doubly fragile” in this scenario. Fourth, among the doubly robust estimators, WReg and CPSW consistently outperformed AIPW in most settings. Fifth, propensity score truncation introduced bias but stabilized estimates. In finite samples, it almost always improved precision and resulted in more accurate estimates of the ATE. Sixth, Hajek weights were always preferred over HT weights for AIPW, as they consistently improved or maintained efficiency. However, for IPW, Hajek weights led to significant efficiency losses when the true OR model was linear (DGM1 and DGM3). Seventh, quadratic specifications of the nuisance models were beneficial for single-model estimators but sometimes worsened the performance of doubly robust estimators. When the true models were linear, quadratic PS models improved IPW efficiency but rarely benefited doubly robust estimators. However, when the true models followed an exponential form, quadratic OR models reduced bias and improved performance for both Reg and doubly robust estimators.

### Simulation study 2

In Simulation Study 2, we adopted the data-generating mechanism from Kang and Schafer (2007) to examine whether the conclusions from Study 1 can be generalized to a different setting.

#### Methods

Let $i = 1, \dots , N$ denote persons for whom realizations $\boldsymbol{x}_i$, $t_i$, and $y_i$ of the random variables $\boldsymbol{X}$, *T*, and *Y* were simulated. The covariate vector $\boldsymbol{X} = (X_1, X_2, X_3, X_4)$ consists of four simulated covariates. A vector of transformed covariates $\widetilde{\boldsymbol{X}}= (\widetilde{{X}}_1, \widetilde{{X}}_2,\widetilde{{X}}_3,\widetilde{{X}}_4) = f ( \boldsymbol{X})$ was defined as

54 $$\begin{aligned} \begin{aligned} \widetilde{x}_{i1}&= 2 \log ( x_{i1} ), \\ \widetilde{x}_{i2}&= ( x_{i2} - 10 )(1+x_{i1}^2 ) , \\ \widetilde{x}_{i3}&= 12.5 ( x_{i3}^{1/3} - 0.6 ) / \log ( x_{i1} ) \text { and} \\ \widetilde{x}_{i4}&= \sqrt{ x_{i4} } - ( x_{i2} - 10 )(1+x_{i1}^2 ) - 20 . \end{aligned} \end{aligned}$$

The components of the vector of transformed covariates $\widetilde{\boldsymbol{X}}$ were independently sampled random variables from a standard normal distribution $\text {N}(0, 1)$, and truncated to the range $[-3, 3]$. This truncation was applied to prevent extreme transformed values resulting from subsequent exponential transformations. The inverse of the transformation in Eq. (54) (i.e., $\boldsymbol{X}= f^{-1} ( \widetilde{\boldsymbol{X}})$) is given by (see Kang and Schafer (2007))

55 $$\begin{aligned} {\begin{matrix} x_{i1} & = \exp \left( \widetilde{x}_{i1} / 2\right) , \\ x_{i2} & = \left( \widetilde{x}_{i2} / \left\{ 1 + \exp (\widetilde{x}_{i1})\right\} \right) + 10, \\ x_{i3} & = \left( \widetilde{x}_{i1} \widetilde{x}_{i3} / 25 + 0.6\right) ^3 \text { and} \\ x_{i4} & = (\widetilde{x}_{i2} + \widetilde{x}_{i4} + 20)^2. \end{matrix}} \end{aligned}$$

It is important to emphasize that in practical applications, analysts only have access to the covariates $\boldsymbol{X}$, while the nonlinear transformation $\widetilde{\boldsymbol{X}}$ remains unknown.

The PS model was defined as

56 $$\begin{aligned} \pi _i = {\text {expit}}\{\alpha _0 + \omega (-\widetilde{x}_{i1} + 0.5 \widetilde{x}_{i2} - 0.25 \widetilde{x}_{i3} - 0.1 \widetilde{x}_{i4})\}, \end{aligned}$$

where $\omega$ was a scaling constant calibrated in a large sample to achieve a desired $R^2$ value for the PS model. The intercept $\alpha _0$ was chosen to ensure the treatment proportion $p_T$ matches the desired value. Treatment realizations $t_i$ were simulated from a Bernoulli distribution with probability $\pi _i$. Note that the PS model involved nonlinearly transformed covariates of observed covariates in the vector $\boldsymbol{x} _i$.

The OR model was also defined as a linear model of nonlinear transformed covariates $\widetilde{\boldsymbol{X}}$

57 $$\begin{aligned} y_i =w (2\widetilde{x}_{i1} + \widetilde{x}_{i2} + \widetilde{x}_{i3} + \widetilde{x}_{i4}) + \varepsilon _i, \end{aligned}$$

where the residual $\varepsilon _i$ was drawn from a normal distribution $\text {N}(0, \sigma ^2)$. The scaling constant *w* and the residual variance $\sigma ^2$ were adjusted to ensure the model attains the desired $R^2$ value and that the overall variance of *Y* equals 1. Because the treatment indicators $t_i$ did not enter the OR model in Eq. (57), the true ATE was 0 in all conditions of Simulation Study 2.

The $R^2$ for both models ranged from 0.25 to 0.5, while the treatment proportion was set at 0.5. For finite-sample performance, sample sizes *N* were set at 500, 1000, and 5000, and for evaluating asymptotic properties, we used $N = 2 \times 10^6$. For each DGMs and each $R^2$ value, we generated 1000 replications for finite-sample analysis (for each $N =$ 500, 1000, and 5000) and 50 replications for asymptotic analysis ($N = 2 \times 10^6$).

As analysis models, we considered two different specifications for the OR and PS models. A correctly specified model used the transformed covariates $\widetilde{\boldsymbol{X}}$ as linear effects, while a misspecified model relied on the untransformed observed covariates $\boldsymbol{X}$ as linear effects. Consequently, four different constellations can be distinguished: (1) OR and PS models are correctly specified, (2) OR model is misspecified, and PS model is correctly specified, (3) OR model is correctly specified, and PS model is misspecified, and (4) both models are misspecified. The same analysis models as in Simulation Study 1 were employed. However, we used only the truncation level 0.01 (i.e., TR1) for the estimators that relied on propensity scores. To quantify the degree of misspecification of the nuisance models, we computed the corresponding $R^2$ values. When the true outcome and propensity score models had $R^2 = 0.25$, the misspecified linear OR and PS models had $R^2 = 0.20$ and 0.23, respectively. When both true models had $R^2 = 0.50$, the corresponding values were $R^2 = 0.40$ and 0.49. This result indicates that the misspecification in the OR models was more pronounced than in the PS models. The evaluation criteria were identical to those used in Simulation Study 1.

**Table 9:**
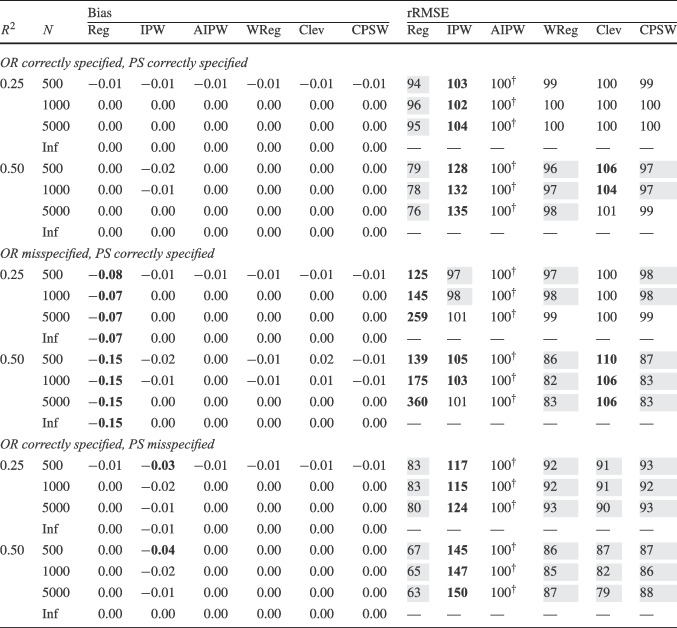
Simulation Study 2: Bias and relative root-mean-square error (rRMSE) of estimators as a function of $R^2$ and sample size (*N*) across different specifications of the OR and PS models.

*Note*. OR = outcome regression; PS = propensity score; Inf = infinite, computed with large samples ($N = 2 \times 10^6$); Reg = regression estimation; IPW = inverse probability weighting; AIPW = augmented inverse probability weighting; WReg = regressions weighted by the inverse of the propensity score; CPSW= calibrated propensity score weighting; Clev = regression with the inverse of the propensity score as a clever covariate. The $R^2$ value applies to both the true OR and true PS models. In analysis models, propensity scores are truncated at [0.01, 0.99], with linear specifications for both OR and PS. For IPW and AIPW, weights are normalized (Hajek type). The rRMSE values are ratios multiplied by 100 for clarity, with AIPW as the reference ($100^\dag$) in each data constellation (specification $\times$ $R^2$ $\times$ *N*). Absolute bias values ($|\text {Bias}|$) $\ge 0.03$ and rRMSE values $\ge 102$ are bolded, while rRMSE values $\le 98$ are shaded in grey

**Table 10:** School meal data example: Descriptive statistics of the variables

			Group mean	
Variable	Meaning	Value	Control	Treatment
BMI	Body mass index	[12.50 - 48.47]	19.82	20.35
School_meal	Participation of school meal programs	(0=no; 1=yes)	0	1
age	Age of child	[4 - 17]	9.85	10.07
ChildSex	Gender of the child	(0=female; 1=male)	0.52	0.51
black	Black race	(0=no; 1=yes)	0.20	0.31
mexam	Hispanic race	(0=no; 1=yes)	0.18	0.33
pir200_plus	Family above 200% of the federal poverty level	(0=no; 1=yes)	0.66	0.25
WIC	Participation in special supplemental nutrition program	(0=no; 1=yes)	0.11	0.26
Food_Stamp	Participation in food stamp program	(0=no; 1=yes)	0.12	0.44
fsdchbi	Childhood food security	(0=insecure; 1=secure)	0.15	0.33
AnyIns	Any insurance	(0=no; 1=yes)	0.89	0.84
RefSex	Gender of the adult respondent	(0=female; 1=male)	0.50	0.39
RefAge	Age of the adult respondent	[18–80]	40.29	38.55

#### Results

Table 9 presents the bias and relative RMSE of the six estimators across four specifications. If both OR and PS models were correctly specified, all estimators were unbiased. Reg was the most accurate, followed by the doubly robust estimators with a slight efficiency loss, while IPW performed the worst. This pattern became more pronounced with larger $R^2$. For $R^2 = 0.5$, WReg and CPSW outperformed AIPW, while Clev exhibited an efficiency loss.

When the OR model was misspecified, and the PS model was correctly specified, Reg was biased, while IPW and doubly robust estimators were unbiased and precise. Doubly robust estimators were generally as good as or better than IPW. However, AIPW performed slightly worse than IPW when $R^2 = 0.25$. Additionally, WReg and CPSW outperformed AIPW, while Clev showed efficiency loss when $R^2 = 0.5$.

When the OR model was correctly specified and the PS model was misspecified, Reg and doubly robust estimators were unbiased, while IPW was slightly biased in small samples ($N = 500$). Reg provided the most accurate estimates, followed by doubly robust estimators with a slight efficiency loss. IPW performed the worst and was unreliable. Among doubly robust estimators, WReg, CPSW, and Clev outperformed AIPW.

When both the OR model and PS model were misspecified, all estimators were biased. IPW showed a small bias in small samples ($N = 500$), but was essentially unbiased in larger samples. AIPW had the worst performance in terms of bias and RMSE, while WReg and CPSW exhibited similar bias and RMSE to Reg. The bias of Clev was more pronounced than that of IPW and WReg.

#### Summary

The key findings from Simulation Study 2 were generally consistent with those from Simulation Study 1. When the models were correctly specified, all estimators were unbiased. Among them, Reg exhibited the lowest RMSE, followed by the doubly robust estimators, which were less efficient, while IPW showed the poorest precision. When the models underlying single-model estimators (i.e., Reg or IPW) were misspecified, the estimators became biased and imprecise. In contrast, the doubly robust estimators remained unbiased and accurate as long as at least one model was correctly specified. Overall, WReg and CPSW outperformed AIPW. However, when both the OR and PS models were misspecified, doubly robust estimators performed worse than Reg and IPW, although WReg and CPSW performed nearly as well as Reg. This finding supports the concern that doubly robust estimators can yield “doubly fragile” estimates of the ATE when both nuisance models are misspecified (Kang & Schafer, 2007; Ding, 2024, p. 157). Taken together with the results from Study 1, two conclusions can be drawn regarding this concern. First, the occurrence of “doubly fragile” estimates for doubly robust estimators appears to be dataset-specific, as this phenomenon did not arise in Simulation Study 1 (Table 5). Second, it seems to be estimator-specific, with only AIPW performing significantly worse than Reg or IPW in Simulation Study 2, while WReg and CPSW performed nearly as well as Reg (Table 9).

## Empirical example: Effect of participation in school meal programs

In this section, we apply the different estimators to a subset of data from the 2007–2008 National Health and Nutrition Examination Surveys (NHANES). This subset has been previously used to illustrate different causal estimators (Chan et al., 2016; Ding, 2024). The dataset has been downloaded from https://doi.org/10.7910/DVN/ZX3VEV (Ding, 2023) and is also available in Supplement D. We are interested in whether participation in school meal programs leads to an increase in body mass index (BMI) among school children. In the supplementary material, we provide two tutorial demonstration documents. The first (Supplement B) is an analysis example of this dataset using the marginaleffects and WeightIt packages, illustrating how analysts can apply R packages to estimate the ATE. The second (Supplement C) presents analyses using all estimators implemented in custom R code, providing a user-friendly demonstration of the underlying mechanisms of each estimator.

The dataset includes 2330 participants, with 1284 ($55.1\%$) in the treatment group (school meal program participants) and 1046 ($44.9\%$) in the control group (non-participants). It contains 13 variables, where “BMI” serves as the outcome variable, and “School_meal” as the treatment variable. The remaining 11 variables are covariates (see Table 10). The mean values of the covariates between the treatment and control groups were unbalanced. Consequently, a naive comparison of BMI means between the two groups could yield biased ATE estimates. To account for differences in covariate distributions, we applied both single-model and doubly robust methods.

**Fig. 1:**
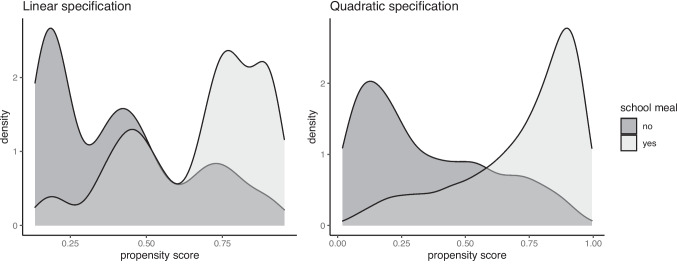
School meal data example: Propensity score distributions under linear and quadratic specifications

**Table 11:** School meal data example: Descriptive statistics of propensity score distributions under linear and quadratic specifications

		Quantile						
	Min	1%	5%	95%	99%	Max	*M*	*SD*
linear PS	0.134	0.138	0.157	0.913	0.936	0.953	0.551	0.258
quadratic PS	0.019	0.029	0.067	0.944	0.972	0.995	0.551	0.303

*Note*. Min = minimum; Max = maximum; *M* = mean; *SD* = standard deviation; PS = propensity score

For the nuisance models, we considered two specifications: a linear specification and a quadratic specification. The quadratic specification incorporated interaction terms and quadratic effects for the two continuous covariates, child’s age and adult respondent’s age. In the OR models, the AIC increased from 7636 (linear) to 7676 (quadratic) in the treatment group, and from 6044 (linear) to 6066 (quadratic) in the control group, indicating that the quadratic specification did not improve model fit. This is further supported by the small changes in $R^2$ values, which increased only slightly from 0.26 to 0.30 in the treatment group, and from 0.36 to 0.41 in the control group. In contrast, for the PS model, the quadratic specification improved model fit, as reflected by a decrease in AIC from 2546 to 2361 and a notable increase in $R^2$ from 0.33 to 0.47.

**Table 12:** School meal data example: Estimated average treatment effect and standard errors of different estimators

	Linear			Quadratic		
Estimator	TR0	TR1	TR5	TR0	TR1	TR5
Reg	−0.0170 (0.2278)	—	—	−0.1942 (0.2381)	—	—
IPW HT	−1.5157 (0.5049)	−1.5157 (0.5049)	−1.4985 (0.4719)	0.7035 (1.7537)	0.7035 (1.1187)	0.6524 (0.6460)
IPW Hajek	−0.1556 (0.2485)	−0.1556 (0.2485)	−0.1515 (0.2471)	−0.4427 (0.5148)	−0.4427 (0.3733)	−0.3911 (0.2608)
AIPW HT	−0.0193 (0.2336)	−0.0193 (0.2336)	−0.0189 (0.2341)	−0.4142 (0.3744)	−0.4142 (0.2792)	−0.3404 (0.2408)
AIPW Hajek	−0.0253 (0.2327)	−0.0253 (0.2327)	−0.0248 (0.2332)	−0.4167 (0.3298)	−0.4167 (0.2776)	−0.3430 (0.2416)
Wreg	−0.0610 (0.2297)	−0.0610 (0.2297)	−0.0602 (0.2300)	−0.2794 (0.2418)	−0.2794 (0.2418)	−0.2764 (0.2413)
Clev	−0.0191 (0.2322)	−0.0191 (0.2322)	−0.0187 (0.2327)	−0.4297 (0.2674)	−0.4281 (0.2620)	−0.3582 (0.2438)
CPSW	−0.0605 (0.2296)	−0.0605 (0.2296)	−0.0597 (0.2300)	−0.3143 (0.2373)	−0.3143 (0.2371)	−0.3005 (0.2377)

*Note*. Sample size = 2330; linear = both analysis models for outcome and treatment were specified linearly; quadratic = both analysis models were specified with quadratic and interaction terms; TR0 = propensity score truncation at [0.0001, 0.9999]; TR1 = [0.01, 0.99]; TR5 = [0.05, 0.95]; Reg = regression estimation; IPW = inverse probability weighting; HT = unnormalized weights (i.e., Horvitz-Thompson); Hajek = normalized weights; AIPW = augmented inverse probability weighting; WReg = regressions weighted by the inverse of the propensity score; Clev = regression with the inverse of the propensity score as a clever covariate; CPSW= calibrated propensity score weighting; inverse propensity scores were used as initial weights in calibrated propensity score weighting; estimated average treatment effects are shown without parentheses; standard errors are shown in parentheses; standard errors were obtained by 1000 bootstrap samples; propensity score truncation is irrelevant to Reg

Figure 1 presents density plots of the estimated propensity scores for the linear (left panel) and quadratic (right panel) specifications. The statistics of the distributions are provided in Table 11. As illustrated, the quadratic specification yielded more extreme propensity score estimates, with values ranging from 0.134 to 0.993 in the linear specification and from 0.019 to 0.995 in the quadratic specification. To address extreme values, we implemented three levels of propensity score truncation (TR0, TR1, and TR5) for estimators that rely on the propensity score. Standard errors were computed via nonparametric bootstrap with 1000 resamples, defined as the standard deviation of the ATE estimates across bootstrap samples (Ding, 2024). Standard errors for differences between two ATE estimators were also assessed by calculating the difference in each bootstrap sample. Alternatively, standard errors could be computed using M-estimation methods (Uysal, 2024).

The main results are summarized in Table 12. First, although not shown in the table, the naive estimator—defined as the unadjusted difference in mean outcomes between the treatment and control groups—yielded an estimated ATE of 0.534 ($SE = 0.232$). This suggests that, on average, students who participated in the school meal program had a higher BMI than those who did not. However, after adjusting for covariates using regression estimation (Reg) or any of the four doubly robust methods under a linear specification of the nuisance models, the estimated effect of program participation was substantially reduced and became statistically indistinguishable from zero. Second, the IPW estimates were highly sensitive to the choice of weighting method. Specifically, estimates based on HT weights (ranging between $-1.516$ and $-1.499$) differed considerably from those using Hajek weights (ranging between $-0.156$ and $-0.152$). For example, under truncation level TR1, the estimated difference between the Hajek and HT specifications was $-1.360$ ($SE=0.429$), a statistically significant difference ($p = 0.002$). This highlights the instability of IPW with HT weights (Ding, 2024). Notably, the choice between HT and Hajek weights had little effect on the estimates for AIPW. Third, varying the propensity score truncation level had minimal impact on the ATE estimates under the linear specification. However, this effect became more pronounced under the quadratic specification, likely due to the presence of more extreme propensity scores (see Table 11). More specifically, the frequency of PS values outside the range [0.05, 0.95] increased from four under the linear specification to 159 cases under the quadratic specification. Fourth, under the quadratic specification of the nuisance models, ATE estimates shifted considerably, particularly for IPW with HT weights. For example, under truncation level TR1, the ATE estimates changed from $-1.516$ with the linear PS model to 0.704 with the quadratic PS model, resulting in a difference of $-2.219$ ($SE=1.199$, $p=0.065$). Overall, estimates of Reg, IPW with Hajek weights, and the four doubly robust estimators became more negative under the quadratic specification but remained statistically indistinguishable from zero. Furthermore, standard error estimates for all methods generally increased under the nuisance model’s quadratic specification. In summary, across various estimation approaches (except for IPW with HT weights), the results consistently indicate that participation in the school meal program had no statistically significant effect on students’ BMI.

## Discussion

In this paper, we compared several doubly robust estimators for the ATE with two widely used single-model methods (regression estimation and IPW) that are commonly applied in psychological research. Our simulation studies highlighted several advantages of doubly robust estimators. When the outcome-covariate relationship was correctly specified, doubly robust methods exhibited only minor efficiency losses compared to regression estimation. However, when either the outcome model or the propensity score model was misspecified, doubly robust estimators remained unbiased, whereas single-model estimators became biased. Even when both the outcome and propensity score models were misspecified, doubly robust methods outperformed single-model estimators in several scenarios. Nevertheless, as demonstrated in Simulation Study 2, certain dataset-specific conditions can make doubly robust estimators doubly fragile, leading single-model estimators to produce more accurate ATE estimates (i.e., they have a lower RMSE). Overall, the primary advantage of doubly robust estimators is their ability to yield consistent ATE estimates even when at most one of the two models is misspecified, offering analysts a second chance to obtain an unbiased estimate.

We also examined different analytic choices for ATE estimation. Our results highlighted the benefits of propensity score truncation for stabilizing estimates, with an optimal truncation level balancing bias and variance. Additionally, normalized Hajek-type weights consistently outperformed or performed at least as well as HT weights for the AIPW estimator, making them the preferred choice. Finally, incorporating more complex model specifications–such as quadratic and interaction terms–enhanced ATE estimator performance in scenarios with complex relationships between the outcome and covariates or between treatment assignment and covariates. However, as with all simulation studies, the conclusions drawn from our simulations are limited to the conditions considered and are not intended to exhaustively characterize the performance of doubly robust methods across all possible data-generating processes (and doing so is not feasible). Further research is necessary to determine the circumstances under which doubly robust estimators produce “doubly fragile” estimates when both the propensity score and outcome models are misspecified (Cao et al., 2009; Tan, 2010; Vermeulen & Vansteelandt, 2015).

Among the various doubly robust estimators, regression weighted by the inverse of the propensity scores (WReg) demonstrated strong performance. It consistently outperformed AIPW and was never inferior to regression with the inverse of the propensity score as a clever covariate (Clev) or calibrated propensity score weighting (CPSW). This aligns with recent recommendations (Gabriel et al., 2024), highlighting regression weighted by the inverse of the propensity scores as a promising doubly robust method. Not only is it easy to implement in standard software, but it is also readily extendable to other models, such as those for categorical outcomes or longitudinal treatment effects (Arel-Bundock et al., 2024). One key reason for its strong performance is that while the regression coefficients are estimated using data weighted by the inverse of the propensity score, the ATE itself is computed from unweighted data. This approach mitigates the risk of instability in the estimates, making regression weighted by the inverse of the propensity scores a particularly appealing choice for practical applications. However, some methods that combine outcome models with propensity scores were not covered in this paper. One such approach is propensity score matching, in which individuals with similar propensity scores from the treatment and control groups are matched. A regression analysis is then conducted on the matched sample to further adjust for any remaining imbalance between groups. A key limitation of this method is that, in small to moderate sample sizes, propensity score matching can lead to efficiency losses due to the exclusion of individuals without suitable matches in the opposite group (Schafer & Kang, 2008). Another approach is to include the propensity score as an additional covariate in the outcome model. However, this method is generally not doubly robust and only produces an unbiased ATE estimate under the assumption of a constant treatment effect (i.e., no treatment-by-covariate interactions). Furthermore, it is crucial to allow for a flexible functional form when modeling the relationship between propensity scores and the outcome (Little & An, 2004). In unreported simulations, we found that this approach was generally inferior to the other doubly robust methods. Similarly, Gabriel et al. (2024) does not consider regression with the propensity scores as an additional covariate to be a truly doubly robust method (see also Ding, 2024).

In this study, we focus on the ATE, as it is the estimand most commonly considered in psychological research. Nevertheless, doubly robust methods can be extended to alternative estimands that target treatment effects for specific subpopulations (for an overview of different estimands, see Greifer & Stuart, 2021). The average treatment effect on the treated (ATT) and average treatment effect on the controls (ATC) restrict attention to the treated and control groups, respectively. When these estimands differ from the ATE, it reflects treatment-effect heterogeneity between groups. For the ATT, only treated units are of interest. Their observed outcomes correspond to treated potential outcomes, and only their counterfactual outcomes under control $\textsf{E}(Y_i(0) \mid \boldsymbol{X}_i, T_i = 1)$ need to be recovered. Consequently, identification relies on ignorability and positivity conditions that apply only to the control potential outcomes, leading to one-sided identification assumptions, namely $Y(0) \perp T \mid \boldsymbol{X}$ and $\pi (\boldsymbol{X}) < 1$ (Ding, 2024). From an estimation perspective, the treated potential outcomes $Y_i(1)$ are observed for treated units, only the outcome model for the control group, $\mu _0(\boldsymbol{X})$, needs to be specified and estimated. This model is then used to predict counterfactual outcomes for treated units. In parallel, inverse propensity score weighting assigns unit weights ($W_i = 1$) to treated observations, while control observations are reweighted by $W_i = \pi (\boldsymbol{X}_i, \hat{\boldsymbol{\alpha }}) / \{1 - \pi (\boldsymbol{X}_i, \hat{\boldsymbol{\alpha }})\}$ to represent the treated population (Li et al., 2018). Doubly robust estimators for the ATT combine these two components to approximate $\textsf{E}\left( Y_i(0) \mid \boldsymbol{X}_i, T_i = 1\right)$. As a result, asymptotic unbiasedness is retained if either the propensity score model or the control outcome model is correctly specified (Ding, 2024; Moodie et al., 2018). The same logic applies to the ATC, with the roles of treated and control units reversed. In the online supplementary materials (Supplement B), we show how the ATT and ATC can be estimated for the school meal data example using R packages. More generally, treatment effects may vary with covariates, giving rise to the conditional average treatment effect (CATE) and, more broadly, treatment effect heterogeneity (Arel-Bundock, 2026). Doubly robust approaches for estimating CATEs have also been developed (e.g., Kennedy, 2023).

In our discussion of the different ATE estimators, we focused on point estimation and did not evaluate the performance of variance estimators. In general, standard errors can be obtained by either M-estimation or bootstrap (Arel-Bundock, 2026, Ch. 14). On the one hand, previous studies derive variance estimators based on M-estimation for ATE estimators (Gabriel et al., 2024; Hansen & Overgaard, 2025; Lok, 2024; Lunceford & Davidian, 2004; Schafer & Kang, 2008). M-estimation theory yields an estimator of the variance matrix of joint model parameters; that is, the regression parameters in the propensity score model and the outcome models. The variances of the different ATE estimators are (nonlinear) functions of these joint model parameters, obtained by applying the delta method. It is important to point out that in this approach, the uncertainty arising from propensity score estimation as well as sampling variability in outcome models is accounted for, thus providing asymptotically valid standard errors (Greifer, 2025).

On the other hand, the bootstrap is also widely recommended for standard error estimation of treatment effects (Ding, 2024; Funk et al., 2011; Glynn & Quinn, 2010; Imbens, 2004). The bootstrap provides a straightforward way to quantify uncertainty for the ATE estimators introduced in this paper without requiring analytic variance derivations for each estimator. It generally yields accurate variance estimates under standard regularity conditions (Austin, 2016, 2022). Because the bootstrap is a unified and broadly applicable method for estimating standard errors for all ATE estimators considered in this paper, we use bootstrap as the default method for standard error estimation in our data example.

The findings of our study open several directions for future research. First, our simulation results indicate that truncating propensity score values can significantly improve the finite-sample performance of weighting-based estimators of the ATE. Further exploration of data-driven approaches, such as propensity score shrinking (Pohlmeier et al., 2016) or data-driven choice of truncation values (Gruber et al., 2022; van der Laan et al., 2024), could help determine the optimal truncation level for specific applications. Second, while this study focused on estimating the effects of binary treatments, many psychological research applications involve assessing continuous exposure variables (e.g., the effects of instructional quality on learning or exercise on health outcomes; see also Lüdtke & Robitzsch, 2025). ATEs for continuous treatments are typically defined as one-dimensional summaries of the treatment effect function, which is also known as the dose-response function (Vansteelandt & Dukes, 2022). A key challenge is that there are different ways to summarize the causal effect for continuous treatments. Future research should further explore the application of doubly robust methods in this context (Kennedy, 2019; Kennedy et al., 2017). Third, we assumed that covariates were measured without error. However, in practical applications, measurement error is common and can bias ATE estimates by distorting covariate adjustment (Sengewald et al., 2019). Future studies should investigate the integration of measurement error correction methods into doubly robust estimation approaches (McCaffrey et al., 2013). Finally, this paper focused on doubly robust approaches using parametric nuisance models with known functional forms. However, growing interest in machine learning-based doubly robust methods has led to the development of semiparametric and nonparametric approaches that estimate the functional form of the outcome and propensity score models in a data-driven way (Chernozhukov et al., 2018; Luque-Fernandez et al., 2018; van der Laan & Rose, 2011). Future research should examine these computationally intensive techniques and compare their performance with parametric modeling strategies (Naimi et al., 2023).

## Data Availability

The dataset used in the empirical example is available in Supplement D at https://osf.io/6veaj/.

## Footnotes

1 It should be added that ATE can also be obtained as an estimate of the coefficient of *T* in a regression model that includes the centered covariates and their interaction terms with *T* (Lin, 2013). However, it is important that in the case of nonlinear effects, also the higher-order terms (e.g., $x_{1i}^2$) need to be centered at their respective means (e.g., $\overline{x_{1}^2}$)

2 In a small number of scenarios under DGM2 with $p_T = 0.3$, IPW slightly outperforms AIPW in terms of precision (see Tables 3, 6, and 7). This may occur because misspecification of the OR component can inflate the variance of the augmentation term in AIPW when treatment assignment is unbalanced. Importantly, this behavior is not systematic, and AIPW remains more precise than IPW in the majority of simulation settings.

## Code availability

R source code to reproduce the results is available in Supplement D at https://osf.io/6veaj/.

## Declarations

### Open Practices Statement

All supplementary materials (Supplement A to D) are available at https://osf.io/6veaj/. The data in the simulation studies can be fully reproduced using the provided code in Supplement D. None of the results reported were preregistered.

## References

[CR1] Arel-Bundock, V. (2026). Model to meaning: How to interpret statistical models with R and Python. *CHAPMAN & HALL CRC*. 10.1201/9781003560333

[CR2] Arel-Bundock, V., Greifer, N., & Heiss, A. (2024). How to interpret statistical models using marginaleffects for R and Python. *Journal of Statistical Software,**111*(9), 1–32. 10.18637/jss.v111.i09

[CR3] Austin, P. C. (2011). An introduction to propensity score methods for reducing the effects of confounding in observational studies. *Multivariate Behavioral Research,**46*(3), 399–424. 10.1080/00273171.2011.568786 21818162 10.1080/00273171.2011.568786 PMC3144483

[CR4] Austin, P. C. (2016). Variance estimation when using inverse probability of treatment weighting (IPTW) with survival analysis. *Statistics in Medicine,**35*(30), 5642–5655. 10.1002/sim.7084 27549016 10.1002/sim.7084 PMC5157758

[CR5] Austin, P. C. (2022). Bootstrap vs asymptotic variance estimation when using propensity score weighting with continuous and binary outcomes. *Statistics in Medicine,**41*(22), 4426–4443. 10.1002/sim.9519 35841200 10.1002/sim.9519 PMC9544125

[CR6] Bang, H., & Robins, J. M. (2005). Doubly robust estimation in missing data and causal inference models. *Biometrics,**61*(4), 962–973. 10.1111/j.1541-0420.2005.00377.x 16401269 10.1111/j.1541-0420.2005.00377.x

[CR7] Brumback, B. A. (2022). Fundamentals of causal inference with R. *Chapman and Hall/CRC*. 10.1201/9781003146674

[CR8] Cao, W., Tsiatis, A. A., & Davidian, M. (2009). Improving efficiency and robustness of the doubly robust estimator for a population mean with incomplete data. *Biometrika,**96*(3), 723–734. 10.1093/biomet/asp033 20161511 10.1093/biomet/asp033 PMC2798744

[CR9] Chan, K. C. G., Yam, S. C. P., & Zhang, Z. (2016). Globally efficient non-parametric inference of average treatment effects by empirical balancing calibration weighting. *Journal of the Royal Statistical Society Series B: Statistical Methodology,**78*(3), 673–700. 10.1111/rssb.12129 27346982 10.1111/rssb.12129 PMC4915747

[CR10] Chattopadhyay, A., Hase, C. H., & Zubizarreta, J. R. (2020). Balancing vs modeling approaches to weighting in practice. *Statistics in Medicine,**39*(24), 3227–3254. 10.1002/sim.8659 32882755 10.1002/sim.8659

[CR11] Chernozhukov, V., Chetverikov, D., Demirer, M., Duflo, E., Hansen, C., Newey, W., & Robins, J. M. (2018). Double/debiased machine learning for treatment and structural parameters. *The Econometrics Journal,**21*(1), C1–C68. 10.1111/ectj.12097

[CR12] Cole, S. R., & Hernán, M. A. (2008). Constructing inverse probability weights for marginal structural models. *American Journal of Epidemiology,**168*(6), 656–664. 10.1093/aje/kwn164 18682488 10.1093/aje/kwn164 PMC2732954

[CR13] Dehejia, R. H., & Wahba, S. (2002). Propensity score-matching methods for nonexperimental causal studies. *Review of Economics and Statistics,**84*(1), 151–161. 10.1162/003465302317331982

[CR14] Deshpande, S., & Kuleshov, V. (2023). Calibrated and conformal propensity scores for causal effect estimation. arXiv:2306.00382. 10.48550/arXiv.2306.00382 PMC12395260 40896453

[CR15] Ding, P. (2023). Replication data for: A first course in causal inference. *Harvard Dataverse*. 10.7910/DVN/ZX3VEV

[CR16] Ding, P. (2024). A first course in causal inference. *Chapman and Hall/CRC*. 10.1201/9781003484080

[CR17] Ellul, S., Carlin, J. B., Vansteelandt, S., & Moreno-Betancur, M. (2024). Causal machine learning methods and use of sample splitting in settings with high-dimensional confounding. arXiv:2405.15242. https://doi.org/10.48550/arXiv.2405.15242 10.1002/sim.70272 PMC12457817 40988329

[CR18] Fan, J., Imai, K., Lee, I., Liu, H., Ning, Y., & Yang, X. (2023). Optimal covariate balancing conditions in propensity score estimation. *Journal of Business & Economic Statistics,**41*(1), 97–110. 10.1080/07350015.2021.2002159

[CR19] Freedman, D. A., & Berk, R. A. (2008). Weighting regressions by propensity scores. *Evaluation Review,**32*(4), 392–409. 10.1177/0193841X08317586 18591709 10.1177/0193841X08317586

[CR20] Fuentes, A., Lüdtke, O., & Robitzsch, A. (2022). Causal inference with multilevel data: A comparison of different propensity score weighting approaches. *Multivariate Behavioral Research,**57*(6), 916–939. 10.1080/00273171.2021.1925521 34128730 10.1080/00273171.2021.1925521

[CR21] Funk, M. J., Westreich, D., Wiesen, C., Stürmer, T., Brookhart, M. A., & Davidian, M. (2011). Doubly robust estimation of causal effects. *American Journal of Epidemiology,**173*(7), 761–767. 10.1093/aje/kwq439 21385832 10.1093/aje/kwq439 PMC3070495

[CR22] Gabriel, E. E., Sachs, M. C., Martinussen, T., Waernbaum, I., Goetghebeur, E., Vansteelandt, S., & Sjölander, A. (2024). Inverse probability of treatment weighting with generalized linear outcome models for doubly robust estimation. *Statistics in Medicine,**43*(3), 534–547. 10.1002/sim.9969 38096856 10.1002/sim.9969

[CR23] Gao, M., & Ding, P. (2023). Causal inference in network experiments: Regression-based analysis and design-based properties. arXiv:2309.07476. 10.48550/arXiv.2309.07476

[CR24] Gelman, A., Jakulin, A., Pittau, M. G., & Su, Y.-S. (2008). A weakly informative default prior distribution for logistic and other regression models. *The Annals of Applied Statistics,**2*(4), 1360–1383. 10.1214/08-AOAS191

[CR25] Gelman, A., & Su, Y.-S. (2024). arm: Data analysis using regression and multilevel/hierarchical models. 10.32614/CRAN.package.arm. R package version 1.14-4

[CR26] Glynn, A. N., & Quinn, K. M. (2010). An introduction to the augmented inverse propensity weighted estimator. *Political Analysis,**18*(1), 36–56. 10.1093/pan/mpp036

[CR27] Graham, B. S., De Xavier Pinto, C. C., & Egel, D. (2012). Inverse probability tilting for moment condition models with missing data. *The Review of Economic Studies,**79*(3), 1053–1079. 10.1093/restud/rdr047

[CR28] Greifer, N. (2025). Estimating effects after weighting. https://ngreifer.github.io/WeightIt/articles/estimating-effects.html

[CR29] Greifer, N., & Stuart, E. A. (2021). Choosing the causal estimand for propensity score analysis of observational studies. arXiv. 10.48550/ARXIV.2106.10577

[CR30] Gruber, S., Phillips, R. V., Lee, H., & van der Laan, M. J. (2022). Data-adaptive selection of the propensity score truncation level for inverse-probability-weighted and targeted maximum likelihood estimators of marginal point treatment effects. *American Journal of Epidemiology,**191*(9), 1640–1651. 10.1093/aje/kwac087 35512316 10.1093/aje/kwac087

[CR31] Gruber, S., & van der Laan, M. J. (2010). An application of collaborative targeted maximum likelihood estimation in causal inference and genomics. *The International Journal of Biostatistics,**6*(1), 18. 10.2202/1557-4679.1182 21731530 10.2202/1557-4679.1182 PMC3126668

[CR32] Gruber, S., & van der Laan, M. J. (2010). A targeted maximum likelihood estimator of a causal effect on a bounded continuous outcome. *The International Journal of Biostatistics,**6*(1), 26. 10.2202/1557-4679.1260 21731529 10.2202/1557-4679.1260 PMC3126669

[CR33] Gruber, S., & van der Laan, M. J. (2012). tmle: An R package for targeted maximum likelihood estimation. *Journal of Statistical Software,**51*(13), 1–35. 10.18637/jss.v051.i1323504300

[CR34] Hahn, J. (1998). On the role of the propensity score in efficient semiparametric estimation of average treatment effects. *Econometrica,**66*(2), 315–331. 10.2307/2998560

[CR35] Hainmueller, J. (2012). Entropy balancing for causal effects: A multivariate reweighting method to produce balanced samples in observational studies. *Political Analysis,**20*(1), 25–46. 10.1093/pan/mpr025

[CR36] Hájek, J. (1971). Comment on "an essay on the logical foundations of survey sampling, part one". In V. P. Godambe & D. A. Sprott (Eds.), *Foundations of statistical inference* (p. 236). Holt, Rinehart, Winston of Canada.

[CR37] Hansen, S. N., & Overgaard, M. (2025). Variance estimation for average treatment effects estimated by g-computation. *Metrika,**88*(4), 419–443. 10.1007/s00184-024-00962-4

[CR38] Hernán, M. A., & Robins, J. M. (2020). Causal inference: What if. Chapman & Hall/CRC Press. https://miguelhernan.org/whatifbook.

[CR39] Hirano, K., & Imbens, G. W. (2001). Estimation of causal effects using propensity score weighting: An application to data on right heart catheterization. *Health Services and Outcomes Research Methodology,**2*(3/4), 259–278. 10.1023/A:1020371312283

[CR40] Hoffmann, N. I. (2023). Double robust, flexible adjustment methods for causal inference: An overview and an evaluation. *SocArXiv*. 10.31235/osf.io/dzayg

[CR41] Holland, P. W. (1986). Statistics and causal inference. *Journal of the American Statistical Association,**81*(396), 945–960. 10.1080/01621459.1986.10478354

[CR42] Horvitz, D. G., & Thompson, D. J. (1952). A generalization of sampling without replacement from a finite universe. *Journal of the American Statistical Association,**47*(260), 663–685. 10.1080/01621459.1952.10483446

[CR43] Imai, K., & Ratkovic, M. (2014). Covariate balancing propensity score. *Journal of the Royal Statistical Society Series B: Statistical Methodology,**76*(1), 243–263. 10.1111/rssb.12027

[CR44] Imbens, G. W. (2004). Nonparametric estimation of average treatment effects under exogeneity: A review. *Review of Economics and Statistics,**86*(1), 4–29. 10.1162/003465304323023651

[CR45] Imbens, G. W., & Rubin, D. B. (2015). Causal inference for statistics, social and biomedical sciences: An introduction. *Cambridge University Press*. 10.1017/CBO9781139025751

[CR46] Ju, C., Schwab, J., & van der Laan, M. J. (2019). On adaptive propensity score truncation in causal inference. *Statistical Methods in Medical Research,**28*(6), 1741–1760. 10.1177/0962280218774817 29991330 10.1177/0962280218774817

[CR47] Källberg, D., & Waernbaum, I. (2023). Large sample properties of entropy balancing estimators of average causal effects. Econometrics and Statistics, Epub ahead of print. 10.1016/j.ecosta.2023.11.004.

[CR48] Kang, J. D. Y., & Schafer, J. L. (2007). Demystifying double robustness: A comparison of alternative strategies for estimating a population mean from incomplete data. *Statistical Science,**22*(4), 523–539. 10.1214/07-STS227 10.1214/07-STS227 PMC2397555 18516239

[CR49] Keele, L., Ben-Michael, E., Lenard, M., & Page, L. (2025). Balancing weights for estimating treatment effects in educational studies. *Journal of Research on Educational Effectiveness*, 1–28. 10.1080/19345747.2025.2483775

[CR50] Keller, B., & Branson, Z. (2024). Defining, identifying, and estimating causal effects with the potential outcomes framework: A review for education research. *Asia Pacific Education Review*. 10.1007/s12564-024-09957-2

[CR51] Kennedy, E. H. (2019). Nonparametric causal effects based on incremental propensity score interventions. *Journal of the American Statistical Association,**114*(526), 645–656. 10.1080/01621459.2017.1422737

[CR52] Kennedy, E. H. (2023). Towards optimal doubly robust estimation of heterogeneous causal effects. *Electronic Journal of Statistics,**17*(2), 3008–3049. 10.1214/23-EJS2157

[CR53] Kennedy, E. H., Ma, Z., McHugh, M. D., & Small, D. S. (2017). Non-parametric methods for doubly robust estimation of continuous treatment effects. *Journal of the Royal Statistical Society Series B: Statistical Methodology,**79*(4), 1229–1245. 10.1111/rssb.12212 28989320 10.1111/rssb.12212 PMC5627792

[CR54] Khan, S., & Ugander, J. (2023). Adaptive normalization for IPW estimation. *Journal of Causal Inference,**11*(1), 20220019. 10.1515/jci-2022-0019

[CR55] Kurz, C. F. (2022). Augmented inverse probability weighting and the double robustness property. *Medical Decision Making,**42*(2), 156–167. 10.1177/0272989X211027181 34225519 10.1177/0272989X211027181 PMC8793316

[CR56] Leite, W. L., Stapleton, L. M., & Bettini, E. F. (2019). Propensity score analysis of complex survey data with structural equation modeling: A tutorial with Mplus. *Structural Equation Modeling: A Multidisciplinary Journal,**26*(3), 448–469. 10.1080/10705511.2018.1522591

[CR57] Li, F., Morgan, K. L., & Zaslavsky, A. M. (2018). Balancing covariates via propensity score weighting. *Journal of the American Statistical Association,**113*(521), 390–400. 10.1080/01621459.2016.1260466

[CR58] Lin, W. (2013). Agnostic notes on regression adjustments to experimental data: Reexamining Freedman’s critique. *The Annals of Applied Statistics,**7*(1), 295–318. 10.1214/12-AOAS583

[CR59] Little, R., & An, H. (2004). Robust likelihood-based analysis of multivariate data with missing values. *Statistica Sinica*, *14*(3), 949–968. http://www.jstor.org/stable/24307424.

[CR60] Liu, L., Hudgens, M. G., & Becker-Dreps, S. (2016). On inverse probability-weighted estimators in the presence of interference. *Biometrika,**103*(4), 829–842. 10.1093/biomet/asw047 29422692 10.1093/biomet/asw047 PMC5793685

[CR61] Lok, J. J. (2024). How estimating nuisance parameters can reduce the variance (with consistent variance estimation). *Statistics in Medicine,**43*(23), 4456–4480. 10.1002/sim.10164 39080846 10.1002/sim.10164 PMC11570876

[CR62] Lüdtke, O., & Robitzsch, A. (2025). ANCOVA versus change score for the analysis of two-wave data. *The Journal of Experimental Education,**93*(2), 363–395. 10.1080/00220973.2023.2246187

[CR63] Lunceford, J. K., & Davidian, M. (2004). Stratification and weighting via the propensity score in estimation of causal treatment effects: A comparative study. *Statistics in Medicine,**23*(19), 2937–2960. 10.1002/sim.1903 15351954 10.1002/sim.1903

[CR64] Luo, S., Min, J., Li, W., Wang, X., & Geng, Z. (2025). A comparative analysis of different adjustment sets using propensity score based estimators. *Computational Statistics & Data Analysis,**203*, 108079. 10.1016/j.csda.2024.108079

[CR65] Luque-Fernandez, M. A., Schomaker, M., Rachet, B., & Schnitzer, M. E. (2018). Targeted maximum likelihood estimation for a binary treatment: A tutorial. *Statistics in Medicine,**37*(16), 2530–2546. 10.1002/sim.7628 29687470 10.1002/sim.7628 PMC6032875

[CR66] Mayer, A., Dietzfelbinger, L., Rosseel, Y., & Steyer, R. (2016). The EffectLiteR approach for analyzing average and conditional effects. *Multivariate Behavioral Research,**51*(2–3), 374–391. 10.1080/00273171.2016.1151334 27249048 10.1080/00273171.2016.1151334

[CR67] McCaffrey, D. F., Lockwood, J., & Setodji, C. M. (2013). Inverse probability weighting with error-prone covariates. *Biometrika,**100*(3), 671–680. 10.1093/biomet/ast022 24795484 10.1093/biomet/ast022 PMC4006991

[CR68] Millimet, D. L., & Tchernis, R. (2009). On the specification of propensity scores, with applications to the analysis of trade policies. *Journal of Business & Economic Statistics,**27*(3), 397–415. 10.1198/jbes.2009.06045

[CR69] Moodie, E. E. M., Saarela, O., & Stephens, D. A. (2018). A doubly robust weighting estimator of the average treatment effect on the treated. *Stat,**7*(1), e205. 10.1002/sta4.205

[CR70] Moore, K. L., & van der Laan, M. J. (2009). Covariate adjustment in randomized trials with binary outcomes: Targeted maximum likelihood estimation. *Statistics in Medicine,**28*(1), 39–64. 10.1002/sim.3445 18985634 10.1002/sim.3445 PMC2857590

[CR71] Morgan, S. L., & Winship, C. (2015). Counterfactuals and causal inference: Methods and principles for social research. *Cambridge University Press*. 10.1017/CBO9781107587991

[CR72] Muñoz, I. D., & van der Laan, M. J. (2012). Population intervention causal effects based on stochastic interventions. *Biometrics,**68*(2), 541–549. 10.1111/j.1541-0420.2011.01685.x 21977966 10.1111/j.1541-0420.2011.01685.x PMC4117410

[CR73] Naimi, A. I., Mishler, A. E., & Kennedy, E. H. (2023). Challenges in obtaining valid causal effect estimates with machine learning algorithms. *American Journal of Epidemiology,**192*(9), 1536–1544. 10.1093/aje/kwab201 34268558 10.1093/aje/kwab201 PMC12096307

[CR74] Pang, M., Schuster, T., Filion, K. B., Schnitzer, M. E., Eberg, M., & Platt, R. W. (2016). Effect estimation in point-exposure studies with binary outcomes and high-dimensional covariate data - A comparison of targeted maximum likelihood estimation and inverse probability of treatment weighting. *The International Journal of Biostatistics,**12*(2), 20150034. 10.1515/ijb-2015-0034 10.1515/ijb-2015-0034 PMC5777857 27889705

[CR75] Petersen, M. L., Porter, K. E., Gruber, S., Wang, Y., & van der Laan, M. J. (2012). Diagnosing and responding to violations in the positivity assumption. *Statistical Methods in Medical Research,**21*(1), 31–54. 10.1177/0962280210386207 21030422 10.1177/0962280210386207 PMC4107929

[CR76] Pirracchio, R., Petersen, M. L., & van der Laan, M. J. (2015). Improving propensity score estimators’ robustness to model misspecification using super learner. *American Journal of Epidemiology,**181*(2), 108–119. 10.1093/aje/kwu253 25515168 10.1093/aje/kwu253 PMC4351345

[CR77] Pohlmeier, W., Seiberlich, R., & Uysal, S. D. (2016). A simple and successful shrinkage method for weighting estimators of treatment effects. *Computational Statistics & Data Analysis,**100*, 512–525. 10.1016/j.csda.2014.09.015

[CR78] R Core Team. (2023). *R: A language and environment for statistical computing*. Manual. Vienna, Austria. https://www.R-project.org/

[CR79] Robins, J. M., Mark, S. D., & Newey, W. K. (1992). Estimating exposure effects by modelling the expectation of exposure conditional on confounders. *Biometrics,**48*(2), 479–495. 10.2307/2532304 1637973

[CR80] Robins, J. M., Rotnitzky, A., & Zhao, L. P. (1994). Estimation of regression coefficients when some regressors are not always observed. *Journal of the American Statistical Association,**89*(427), 846–866. 10.1080/01621459.1994.10476818

[CR81] Robins, J. M., Sued, M., Lei-Gomez, Q., & Rotnitzky, A. (2007). Comment: Performance of double-robust estimators when “inverse probability’’ weights are highly variable. *Statistical Science,**22*(4), 544–559. 10.1214/07-STS227D

[CR82] Rosenbaum, P. R., & Rubin, D. B. (1983). The central role of the propensity score in observational studies for causal effects. *Biometrika,**70*(1), 41–55. 10.1093/biomet/70.1.41

[CR83] Rubin, D. B. (1977). Assignment to treatment group on the basis of a covariate. *Journal of Educational Statistics,**2*(1), 1–26. 10.2307/1164933

[CR84] Rubin, D. B. (1978). Bayesian inference for causal effects: The role of randomization. *The Annals of Statistics,**6*(1), 34–58. 10.1214/aos/1176344064

[CR85] Rudolph, K. E., Williams, N. T., Miles, C. H., Antonelli, J., & Diaz, I. (2023). All models are wrong, but which are useful? Comparing parametric and nonparametric estimation of causal effects in finite samples. *Journal of Causal Inference,**11*(1), 20230022. 10.1515/jci-2023-0022

[CR86] Särndal, C.-E., Swensson, B., & Wretman, J. H. (2003). *Model assisted survey sampling*. Berlin: Springer.

[CR87] Schafer, J. L., & Kang, J. D. Y. (2008). Average causal effects from nonrandomized studies: A practical guide and simulated example. *Psychological Methods,**13*(4), 279–313. 10.1037/a0014268 19071996 10.1037/a0014268

[CR88] Scharfstein, D. O., Rotnitzky, A., & Robins, J. M. (1999). Adjusting for nonignorable drop-out using semiparametric nonresponse models: Rejoinder. *Journal of the American Statistical Association,**94*(448), 1135–1146. 10.2307/2669930

[CR89] Schuler, M. S., & Rose, S. (2017). Targeted maximum likelihood estimation for causal inference in observational studies. *American Journal of Epidemiology,**185*(1), 65–73. 10.1093/aje/kww165 27941068 10.1093/aje/kww165

[CR90] Sengewald, M.-A., Steiner, P. M., & Pohl, S. (2019). When does measurement error in covariates impact causal effect estimates? Analytic derivations of different scenarios and an empirical illustration. *British Journal of Mathematical and Statistical Psychology,**72*(2), 244–270. 10.1111/bmsp.12146 30345554 10.1111/bmsp.12146

[CR91] Shah, B. R., Laupacis, A., Hux, J. E., & Austin, P. C. (2005). Propensity score methods gave similar results to traditional regression modeling in observational studies: A systematic review. *Journal of Clinical Epidemiology,**58*(6), 550–559. 10.1016/j.jclinepi.2004.10.016 15878468 10.1016/j.jclinepi.2004.10.016

[CR92] Smith, J. A., & Todd, P. E. (2005). Does matching overcome LaLonde’s critique of nonexperimental estimators? *Journal of Econometrics,**125*(1–2), 305–353. 10.1016/j.jeconom.2004.04.011

[CR93] Tan, Z. (2010). Bounded, efficient and doubly robust estimation with inverse weighting. *Biometrika,**97*(3), 661–682. 10.1093/biomet/asq035

[CR94] Thoemmes, F., & Ong, A. D. (2016). A primer on inverse probability of treatment weighting and marginal structural models. *Emerging Adulthood,**4*(1), 40–59. 10.1177/2167696815621645

[CR95] Uysal, D. (2024). Estimation of causal effects with a binary treatment variable: A unified M-estimation framework. *Journal of Econometric Methods,**13*(1), 145–204. 10.1515/jem-2020-0021

[CR96] van der Laan, L., Lin, Z., Carone, M., & Luedtke, A. (2024). Stabilized inverse probability weighting via isotonic calibration. arXiv, arXiv:2411.06342. 10.48550/arXiv.2411.06342 PMC13061341 41958837

[CR97] van der Laan, M. J., & Rose, S. (2011). *Targeted learning: Causal inference for observational and experimental data*. 10.1007/978-1-4419-9782-1

[CR98] van der Laan, M. J., & Rubin, D. (2006). Targeted maximum likelihood learning. *The International Journal of Biostatistics*, *2*(1). 10.2202/1557-4679.1043

[CR99] Vansteelandt, S., & Dukes, O. (2022). Assumption-lean inference for generalised linear model parameters. *Journal of the Royal Statistical Society Series B: Statistical Methodology,**84*(3), 657–685. 10.1111/rssb.12504

[CR100] Vermeulen, K., & Vansteelandt, S. (2015). Bias-reduced doubly robust estimation. *Journal of the American Statistical Association,**110*(511), 1024–1036. 10.1080/01621459.2014.958155

[CR101] Waernbaum, I., & Pazzagli, L. (2023). Model misspecification and bias for inverse probability weighting estimators of average causal effects. *Biometrical Journal,**65*(2), 2100118. 10.1002/bimj.202100118 10.1002/bimj.202100118 PMC10087564 36045099

[CR102] Wang, L., Zhang, Y., Richardson, T. S., & Zhou, X.-H. (2021). Robust estimation of propensity score weights via subclassification. arXiv:1602.06366. 10.48550/arXiv.1602.06366

[CR103] Yang, S. (2018). Propensity score weighting for causal inference with clustered data. *Journal of Causal Inference,**6*(2), 20170027. 10.1515/jci-2017-0027

[CR104] Zhao, Q., & Percival, D. (2017). Entropy balancing is doubly robust. *Journal of Causal Inference,**5*(1), 20160010. 10.1515/jci-2016-0010

[CR105] Zubizarreta, J. R. (2015). Stable weights that balance covariates for estimation with incomplete outcome data. *Journal of the American Statistical Association,**110*(511), 910–922. 10.1080/01621459.2015.1023805

[CR106] Zubizarreta, J. R., Stuart, E. A., Small, D. S., & Rosenbaum, P. R. (2023). Handbook of matching and weighting adjustments for causal inference. 10.1201/9781003102670

